# Molecular signatures in IASLC/ATS/ERS classified growth patterns of lung adenocarcinoma

**DOI:** 10.1371/journal.pone.0206132

**Published:** 2018-10-23

**Authors:** Heike Zabeck, Hendrik Dienemann, Hans Hoffmann, Joachim Pfannschmidt, Arne Warth, Philipp A. Schnabel, Thomas Muley, Michael Meister, Holger Sültmann, Holger Fröhlich, Ruprecht Kuner, Felix Lasitschka

**Affiliations:** 1 Department of Thoracic Surgery, Thoraxklinik, University Hospital Heidelberg, Heidelberg, Germany; 2 Translational Lung Research Centre Heidelberg (TLRC-H), German Centre for Lung Research (DZL), Heidelberg, Germany; 3 Institute of Pathology, University Hospital Heidelberg, Heidelberg, Germany; 4 Translational Research Unit (STF), Thoraxklinik, University of Heidelberg, Heidelberg, Germany; 5 Cancer Genome Research (B063), German Cancer Research Center (DKFZ) and German Cancer Consortium (DKTK), Heidelberg, Germany; 6 Institute for Computer Science, c/o Bonn-Aachen International Center for IT, Algorithmic Bioinformatics, University of Bonn, Bonn, Germany; Institute for Bioscience and Biotechnology Research, ITALY

## Abstract

**Background:**

The current classification of human lung adenocarcinoma defines five different histological growth patterns within the group of conventional invasive adenocarcinomas. The five growth patterns are characterised by their typical architecture, but also by variable tumor biological behaviour.

**Aims:**

The aim of this study was to identify specific gene signatures of the five adenocarcinoma growth patterns defined by the joint IASLC/ATS/ERS working group.

**Methods:**

Total RNA from microdissected adenocarcinoma tissue samples of ten lepidic, ten acinar, ten solid, nine papillary, and nine micropapillary tumor portions was isolated and prepared for gene expression analysis. Differential expression of genes was determined using the R package “LIMMA”. The overall significance of each signature was assessed via global test. Gene ontology statistics were analysed using GOstat. For immunohistochemical validation, tissue specimens from 20 tumors with solid and 20 tumors with lepidic growth pattern were used.

**Results:**

Microarray analyses between the growth patterns resulted in numerous differentially expressed genes between the solid architecture and other patterns. The comparison of transcriptomic activity in the solid and lepidic patterns revealed 705 up- and 110 downregulated non-redundant genes. The pattern-specific protein expression of *Inositol-1*,*4*,*5-trisphosphate-kinase-A (ITPKA)* and *angiogenin* by immunohistochemistry confirmed the RNA levels. The strongest differences in protein expression between the two patterns were shown for *ITPKA* (p = 0.02) and *angiogenin* (p = 0.113).

**Conclusions:**

In this study growth pattern-specific gene signatures in pulmonary adenocarcinoma were identified and distinct transcriptomic differences between lung adenocarcinoma growth patterns were defined. The study provides valuable new information about pulmonary adenocarcinoma and allows a better assessment of the five adenocarcinoma subgroups.

## Introduction

In 2011, a joint IASLC/ATS/ERS working group introduced a new classification of human lung adenocarcinoma [1]. This new classification defined five different histological growth patterns within the group of conventional invasive adenocarcinomas: lepidic (corresponding to the former non-mucinous bronchioloalveolar pattern), acinar, papillary, micropapillary (newly added) and solid predominant adenocarcinoma. Overall tumor classification was suggested to be done according to the predominant pattern with additional documentation of the percentage distribution of all evident architectures [1,2]. The five growth patterns should be characterised by their typical architecture, but also by variable tumor biological behaviour. The predominant pattern has relevant influence on the disease-free and long-term survival of patients. Whereas lepidic predominant adenocarcinoma seems to be the one with the best prognosis, the micropapillary and solid architectures are associated with a particularly poor prognosis [3–7]. On the molecular level, lung adenocarcinomas have been characterised by diverse transcriptional profiles [8–10]. Three molecular subtypes (bronchoid/terminal respiratory, magnoid/proximal-proliferative and squamoid/proximal-inflammatory) were defined and successfully verified across several microarray and sequencing datasets [11–13]. Such molecular profiles are used to decipher prognostic/predictive biomarkers and therapeutic target sites specific for patient subgroups. However, the present transcriptomic profiles of lung adenocarcinoma do not necessarily reflect the distinct histological architectures. Our study combined tissue microdissection and molecular profiling of the five lung adenocarcinoma growth patterns in order to precisely identify specific genes signatures. Selected biomarkers and drug targeted candidates were validated by immunohistochemistry (IHC) in the lepidic and solid growth patterns.

## Materials and methods

### Patients

Cryopreserved tumor tissue of 48 patients was selected based on the histopathological findings following surgery for lung adenocarcinoma. All tumor sections were analysed for their growth patterns including lepidic (10), acinar (10), papillary (9), micropapillary (9) and solid (10) architecture. Cryopreserved tumor tissue was provided by lung biobank Heidelberg, a member of the biomaterial bank Heidelberg (BMBH) and of the biobank platform of the German Center for Lung Research (DZL) (Ethical Approval Number: 2070/2001). Tumor sections from an additional 20 patients were used for IHC validation. Paraffin tissue was provided by the tissue bank of the National Center for Tumor Diseases (NCT, Heidelberg, Germany) in accordance with the regulations of the tissue bank and the approval of the ethics committee of Heidelberg University (Ethical Approval Numbers: 206/2005 and 207/2005). All data was fully anonymized before we accessed them. All patients gave their written informed consent to have samples from their medical records used in research.

### Laser-capture microdissection

The isolation of tumor areas representing a specific growth pattern was performed by laser capture microdissection (PALM’s LMPC technology, Carl Zeiss Microscopy GmbH, Göttingen, Germany). Fresh frozen tumor samples were cut in 18 μm thick sections and applied to ZEISS Membrane Slides 1.0 PEN (polyethylene naphthalate), baked for 3.5 h at 180° C, irradiated with UV light (312 nm) for 30 min and cooled to -22°C just before use. The tissue sections were stained with 1% cresyl violet (Sigma) for 15 s. Using the RoboLPC method, between 4 and 10 mm^2^ of cells were cut out [14]. The tissue areas of interest were circumscribed electronically under the microscope, cut automatically by cold laser ablation and catapulted into an opaque ZEISS AdhesiveCap 500 by damage-free laser-induced propulsion [15]. The composition (frequencies) of all 5 growth patterns in each of the 48 tumors has been analyzed (Table 1).

**Table 1:** Percentage distribution of growth patterns.

Sample	Microdissected growth pattern	solid	acinar	papillary	micropapillary	lepidic
LC01	acinar	0	75	0	0	25
LC02	acinar	0	80	10	5	5
LC03	acinar	0	80	0	0	20
LC04	acinar	0	95	5	0	0
LC07	acinar	0	90	10	0	0
LC09	acinar	0	70	20	0	10
LC15	acinar	0	80	20	0	0
LC19	acinar	10	70	20	0	0
LC26	acinar	0	95	0	0	5
LC36	acinar	0	25	70	0	5
LC06	lepidic	0	5	0	0	95
LC18	lepidic	80	0	0	5	15
LC23	lepidic	0	30	10	0	60
LC27	lepidic	0	45	30	0	25
LC30	lepidic	0	0	0	5	95
LC32	lepidic	0	0	10	0	90
LC33	lepidic	30	30	0	0	40
LC34	lepidic	0	10	0	0	90
LC43	lepidic	15	10	15	10	50
LC47	lepidic	0	0	0	20	80
LC08	micropapillary	0	0	10	10	80
LC14	micropapillary	0	80	0	20	0
LC29	micropapillary	0	75	5	20	0
LC31	micropapillary	0	80	0	20	0
LC35	micropapillary	5	30	20	15	30
LC45	micropapillary	5	20	5	40	30
LC37	micropapillary	60	0	0	40	0
LC41	micropapillary	0	70	5	5	20
LC42	micropapillary	0	60	30	10	0
LC17	papillary	15	25	40	20	0
LC22	papillary	0	70	10	5	15
LC24	papillary	0	0	60	20	20
LC28	papillary	10	80	10	0	0
LC44	papillary	0	65	5	5	25
LC46	papillary	0	20	20	0	60
LC38	papillary	10	40	10	0	40
LC39	papillary	10	30	20	10	30
LC40	papillary	0	0	80	0	20
LC05	solid	70	20	10	0	0
LC10	solid	95	0	5	0	0
LC11	solid	100	0	0	0	0
LC12	solid	80	20	0	0	0
LC13	solid	100	0	0	0	0
LC16	solid	60	40	0	0	0
LC20	solid	95	5	0	0	0
LC21	solid	40	60	0	0	0
LC25	solid	80	20	0	0	0
LC48	solid	80	20	0	0	0

Table 1 shows the percentage distribution of the 5 growth patterns in 48 fresh frozen tumor samples used for microdissection.

### Microarray experiments

Total RNA from microdissected adenocarcinoma tissue sections was extracted and quantified using the RNeasy protocol (Qiagen, Hilden, Germany) according to the manufacturer’s instructions. For RNA quality assessment the Agilent 2100 Bioanalyzer System together with the Agilent RNA 6000 Nano Kit was used according to the manufacturer’s instructions (Agilent Technologies, Santa Clara, CA, USA). About 20 ng of total RNA was prepared for microarray hybridisation using the MessageAmp^TM^ Premier RNA Amplification Kit (Thermo Fisher Scientific, Waltham, MA, USA) according to the manufacturer’s instructions. Fragmented biotinylated amplificated RNA was hybridised on Illumina HumanHT-12 v4 Expression Bead Chip including more than 47,000 Probes (Illumina, San Diego, CA, USA). Processing of the Illumina microarray data was performed using the opensource pipeline “Lumi” [16]. More specifically, this pipeline comprises background correction, quantile normalization, model based variance stabilization (PMID 18178591) and detection p-value based present/absent calling. For the following analysis only Entrez ID allocated transcripts with a presence call in each sample of at least one of the studied patient subgroups were considered.

The microarray dataset has been deposited, MIAME compliant, into the NCBI Gene Expression Omnibus database (GSE58772).

Molecular subtype assignment was done as previously described: TCGA data was DESeq normalised and reduced to the 5761 genes, which could be mapped to the data studied here [11–13,17]. Datasets were first Blom-transformed and subsequently adjusted by an empirical Bayes approach to allow for an integration of the data studied here and the TCGA data [18,19]. Genes were further reduced to the overlap with the previously reported signature [12]. Based on the resulting 260 genes, an SVM classifier was trained on the TCGA data in order to predict expression subtypes defined in 12 [12]. The prediction performance of the classifier was evaluated via 10-fold cross-validation, yielding an estimated prediction accuracy of the expression subtype of around 90%. The final SVM model was then asked to make predictions for each sample in our dataset. Respective expression subtype predictions (class probabilities) were illustrated by a clustered heatmap.

Differential expression of genes was determined using the R package “LIMMA” [20]. The overall significance of a signature was assessed via a “global test” [21]. The global test is a set based method, which tries to reject the null hypothesis that all genes in set of interest (in our case all signature genes) show no association to a defined clinical outcome or grouping. Gene ontology statistics were analysed using GOstat [22].

### Immunohistochemistry (IHC)

IHC was done for the validation of identified differentially expressed genes. Additional tissue sections from 20 tumors that had been used for the microarray experiment and another 20 specimens from other tumors with solid and lepidic growth patterns were stained with the corresponding antibodies for *ITPKA (inositol-1*,*4*,*5-trisphosphate-3-kinase-A)* (polyclonal anti-rabbit ITPKA, 1/100, Atlas, Stockholm, Sweden), *PFKP (phosphofructokinase*, *platelet)* (polyclonal anti-rabbit PFKP, 1/50, (Thermo Fisher Scientific, Waltham, MA, USA), *ERRFI1 (MIG6*, *mitogen-inducible gene 6)* (polyclonal rabbit anti-ERRFI1, 1/100, Atlas, Stockholm, Sweden) and *angiogenin (ANG)* (polyclonal rabbit anti-angiogenin, 1/100, Abcam, Cambridge, United Kingdom) using an automated staining protocol on the DAKO autostainer (antigen retrieval with citrate buffer pH 6.0). Positive control tissue sections were chosen according to the manufacturers’ antibodies information, i.e. cerebral cortex for *ITPKA*, kidney for *PFKP*, breast cancer for *ERRFI1*, and liver for *angiogenin*. Isotype- and concentration-matched control antibodies (Dako, Hamburg, Germany) served as negative controls.

Semi-quantitative evaluation of protein expression was done using the H-Score method according to Pirker et al [23]. The percentage of tumor cells at different staining intensities was determined by visual assessment at 200-fold magnification, with the score calculated using the formula 1 x (% of 1+ cells) + 2 x (% of 2+ cells) + 3 x (% of 3+ cells) [23,24]. Samples were classified as negative (H-Score 0–50), weakly positive (H-Score 51–100), moderately positive (H-Score 101–200) or strongly positive (H-Score 201–300). The average H-Score values for each growth pattern and each antibody staining were calculated and compared.

## Results

### Molecular profiling of IASLC/ATS/ERS classified growth patterns

In total microdissected tissue sections of 48 specimens were addressed for RNA extraction and microarray experiments. RIN (RNA integrity number) values between 7 and 8 indicate sufficient RNA quality of microdissected tissues (mean RIN 7.5, SD 0.86) for microarray analysis (S1 Fig). Microarray analyses between the five different growth patterns resulted in numerous differentially expressed genes between the solid architecture and other patterns (S1 Table). Only a few solid-independent comparisons (e.g. papillary vs. micropapillary) indicated significant transcriptome differences. In the following, we focused on the gene signature between the solid and lepidic pattern associated with different clinical outcomes. Earlier, we tested all 48 adenocarcinoma transcriptomes according to their similarities to previously reported gene expression subtypes [12]. Supervised classification using 260 informative genes from the Wilkerson signature showed that our 48 samples could be assigned to each of the three classes proximal-inflammatory (PI), proximal-proliferative (PP) and terminal respiratory unit (TRU) with high confidence (S2 Fig). Furthermore, the unsupervised multidimensional scaling plot of the joint expression data from our samples and the TCGA data indicated, that all our samples fell clearly within the distribution of each of the three classes (S3 Fig). Altogether, the solid patterns (90% of specimens) clearly assigned for the PI subtype, acinar patterns (70%) predominantly assigned for the PP and lepidic patterns (70%) for the TRU subtype (S2 Table). However, differentially expressed genes between the microdissected histological patterns differed from previously reported gene signatures in adenocarcinoma subtypes, as highlighted for the solid-lepidic gene signature in the following paragraph.

### Differences in gene expression, cellular processes and signalling pathways in the solid and lepidic patterns

Comparison of the transcriptomic activity between the solid and lepidic patterns revealed 705 up- and 110 downregulated non-redundant genes (FDR (false discovery rate) 5%, fold change >1.5 or < 0.66) (S3 Table). A clear separation of the specimens of both patterns could be confirmed by hierarchical clustering (S4 Fig). Furthermore, only 25 of 815 deregulated genes (3%) overlapped with the reported 506 LAD predictor genes classifying the molecular subtypes [12]. Similarly, further reported sets of differentially expressed genes between intrinsic molecular subtypes displayed a poor overlap (S4 Table).

Gene ontology analysis suggested several biological processes, which are linked to overrepresented, upregulated genes in the solid pattern. Cancer-associated processes included cell motility, proliferation, cell cycle and negative regulation of apoptosis (Table 2).

**Table 2:** Gene ontologies.

GO	GO as name	Genes	Groupcount	Totalcount	Pvalue
GO:0015031	protein transport	mtx1; sdcbp; ap3b1; lgtn; zw10; tomm40; kpna4; fbxo34; kpna6; pttg1ip; vps35; stx3; tpr; srp19; trpc4ap; stx6; mtx2; nup37; nxt1; snapin; kdelr3; sec22b; rab11fip5; clta; xpo1; ctsa; arl6ip1; rab9a; stat1; bcl6; chchd4; arfgap1; gdi1; copb2; mcm3ap; chmp1b; bcl3; rab22a; rab8a; nup205; atg16l1; tmed2; exoc4; stxbp2; vps37c; unc50; sec31a; exoc7; tomm20; ap2m1; sels; aftph	52	866	2,80E-18
GO:0006915	apoptosis	pmaip1; glo1; pdcd6; fadd; mrps30; tubb2c; api5; casp2; ripk2; igfbp3; rnf34; rb1cc1; tnfrsf12a; mcl1; dpf2; rtkn; trib3; becn1; pdcl3; smndc1; map1s; axin1; puf60; sema4d; tnfrsf21; tia1; acvr1; ctnnbl1; litaf; arhgdia; qrich1; raf1; bcl6; atg12; stat1; bag3; ywhaz; dnajb6; bcl3; mrpl41; hspa1a; ikbkg; ube2z; ifih1; dap3; tfdp1; tax1bp1; elmo2; rasa1	49	855	5,85E-16
GO:0006396	RNA processing	imp3; exosc1; bop1; hnrpul1; ints5; utp6; prpf4; ddx56; magoh; nsun2; prpf19; wbp11; ints8; u2af1; sf3b4; rbm5; hnrnpr; sf3b2; prpf3; dkc1; raly; sfrs17a; snrpb2; smndc1; u2af2; rbm22; pabpc1; adar; puf60; fars2; rnps1	31	525	1,65E-10
GO:0008380	RNA splicing	prpf4; wbp11; magoh; prpf19; u2af1; sf3b4; hnrnpr; sf3b2; prpf3; raly; sfrs17a; snrpb2; smndc1; u2af2; rbm22; puf60; pabpc1; rnps1	18	225	6,84E-06
GO:0009615	response to virus	bcl3; becn1; hnrpul1; banf1; isg15; stat1; ifih1; ifnar2; mx2; irf3; xpo1; tbk1	12	98	8,32E-06
GO:0006605	protein targeting	bcl3; srp19; sdcbp; ap3b1; nup205; arl6ip1; bcl6; nxt1; stat1; kpna4; tomm20; tomm40; kpna6; pttg1ip; mcm3ap; tpr; xpo1	17	218	1,80E-05
GO:0000902	cell morphogenesis	nrp1; net1; sdcbp; bcl6; cap1; e2f4; igfbp3; cyfip1; rb1cc1; baiap2l1; tbce; tnfrsf12a; c20orf20; ogfr; top2b; ryk; plxna3; map1s; sema4d; sipa1; smad4; rasa1; dgkd	23	478	3,87E-05
GO:0022008	neurogenesis	nrp1; map2k1; nptn; prpf19; cyfip1; tbce; tnfrsf12a; tubb3; cdk5rap1; eif2b2; top2b; ryk; pxmp3; map1s; eif2b4; plxna3; sema4d; ngrn	18	262	4,41E-05
GO:0022613	ribonucleoprotein complex biogenesis and assembly	imp3; ebna1bp2; exosc1; mtif3; bop1; lgtn; utp6; ddx56; wbp11; eif2b2; dkc1; gnl2; nip7; smndc1; eif2b4; eif4h; eif3b	17	246	6,98E-05
GO:0043066	negative regulation of apoptosis	bcl3; mcl1; glo1; rtkn; acvr1; becn1; hspa1a; api5; arhgdia; bcl6; bag3; rb1cc1; ywhaz; sema4d; tax1bp1; rasa1	16	227	0,0001
GO:0006512	ubiquitin cycle	fbxo28; pcnp; trim33; fbxl11; atg12; isg15; sumo2; zc3hc1; ube2a; map1lc3b; rnf34; prpf19; spsb1; tceb1; march7; ubac1; fbxo18; klhl12; ubr4; rnf167; ube2z; mib2; fbxo6; syvn1	24	549	0,000184
GO:0006606	protein import into nucleus	bcl3; nup205; bcl6; stat1; kpna4; kpna6; pttg1ip; mcm3ap; tpr; xpo1	10	96	0,000189
GO:0007243	protein kinase cascade	oxsr1; litaf; fadd; ripk2; stat1; ifnar2; mapk8ip3; rb1cc1; slc20a1; tfg; atp6ap2; bcl3; ikbkg; map2k1ip1; mib2; irak1; akap11; tbk1	18	376	0,000448
GO:0007249	I-kappaB kinase/NF-kappaB cascade;	bcl3; litaf; fadd; ikbkg; ripk2; stat1; irak1; mib2; slc20a1; tfg; tbk1	11	139	0,00078
GO:0006928	cell motility	actb; nrp1; actr2; acvr1; map2k1; sdcbp; tubb2c; top2b; arhgdia; bcl6; arpc3; plaur; pxmp3; parp9; plxna3; tnfrsf12a; mkln1	17	383	0,00269
GO:0008283	cell proliferation	nrp1; map2k1; mapre1; gnl3; bcl6; e2f4; mdk; raf1; ripk2; crip2; prpf19; rbbp7; myc; capn1; gpc4; sbds; cnot8; ctnnbip1; dkc1; csk; prmt5; hdgf; tfdp1; sipa1; smad4; klf11; col18a1	27	745	0,00327
GO:0007049	cell cycle	acvr1; map2k1; mapre1; pcnp; zw10; bcl6; e2f4; zc3hc1; ckap5; mapk6; rb1cc1; rbbp8; supt5h; tubb3; cdk5rap1; myc; rbm5; mrpl41; krt7; prmt5; hcfc1; rabgap1; ppp1cb; tfdp1; axin1; hbp1; sipa1; tusc4; rpa1	29	839	0,00484

Table 2 shows gene ontologies for 710 significantly upregulated genes in solid compared with lepidic architecture using GOstat p-value ≤ 0.005. No significant gene ontologies were resulted for the 105 downregulated genes.

Gene expression regulation was represented by RNA processing and splicing. Protein linked processes included ontologies like protein transport, protein targeting, ribonucleoprotein complex biogenesis and ubiquitin cycle. Focusing on signal transduction pathways, members of the MAPK [mitogen-activated protein kinases) signalling (*MAP2K1*, *MAPK6*, *MAPKAPK5*, *MAP2K1IP1* and *MAPK8IP3*) and NF-κB (nuclear factor 'kappa-light-chain-enhancer' of activated B-cells) signalling (*IKBKG*, *LITAF*, *STAT1*, *BCL3*, *TFG* and *TBK1*) were upregulated in the solid pattern.

Independent on DEseq and gene ontology analysis, known oncogenes and tumor suppressors in lung adenocarcinoma have been investigated for gene expression variance. Most of the genes were not informative on transcript level, only MET and MAP2K1 expression indicates upregulation in the solid pattern (S5 Table).

For IHC validation, we selected *PFKP*, *ITPKA* and *ERRFI1* upregulated in the solid pattern and *ANG* upregulated in the lepidic pattern as putative novel biomarkers in distinct predominant architectures.

### Immunohistochemical validation

**Fig 1:**
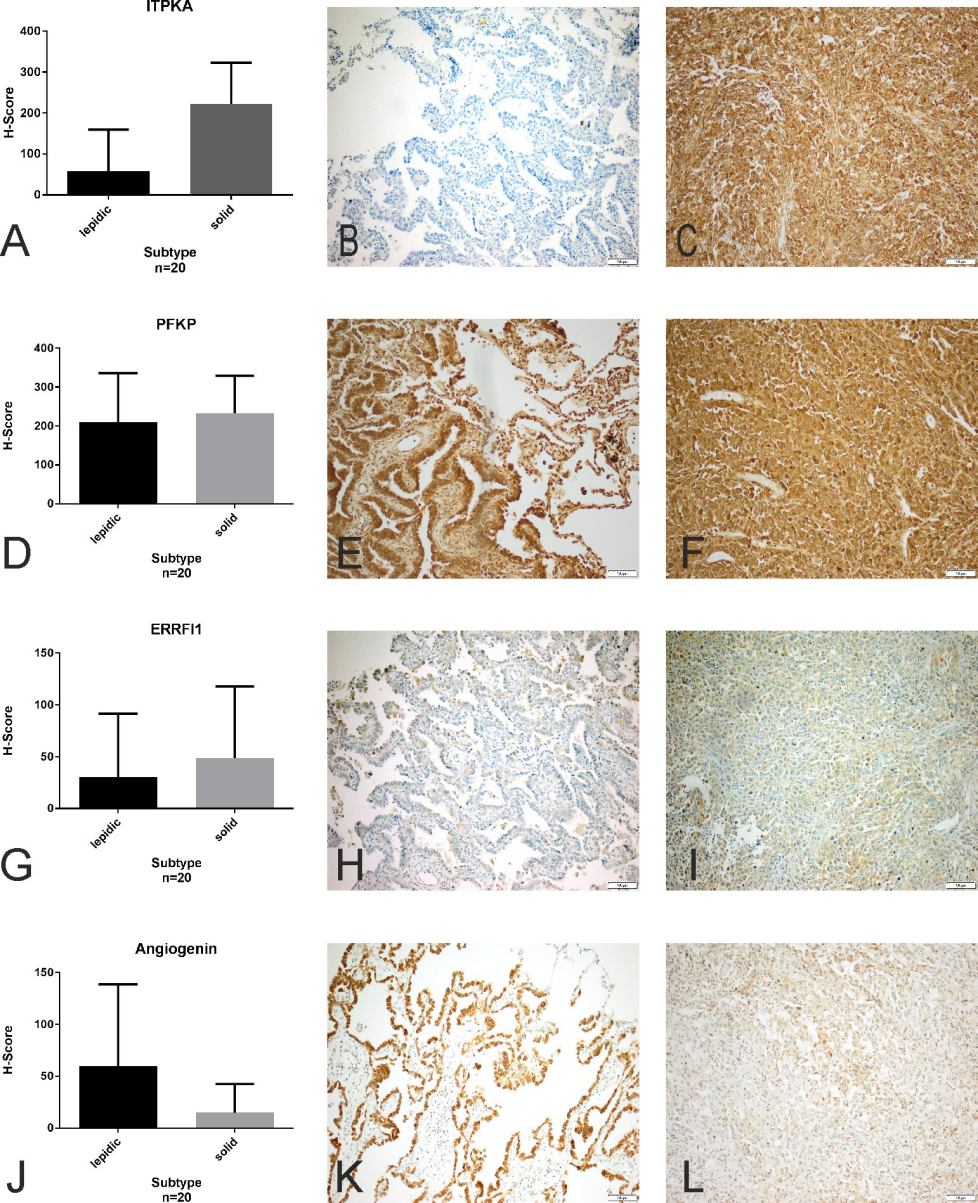
Immunohistochemistry. Immunohistochemical staining of *inositol-1*,*4*,*5-trisphosphate-3-kinase-A (ITPKA)*, *phosphofructokinase*, *platelet (PFKP)*, *mitogen-inducible gene 6 (ERRFI1)* and *angiogenin (ANG)* in pulmonary adenocarcinoma with predominant lepidic (B, E, H, K) and solid growth patterns (C, F, I, L). 200x magnification. Histograms depict mean staining scores with standard deviation of n = 20 adenocarcinomas each (A, D, G, J).

The pattern-specific protein expression of *ITPKA* and *angiogenin* by immunohistochemistry confirmed the RNA levels. The protein *ITPKA* was more abundant in the solid pattern, showing cytoplasmic staining, and *angiogenin* was more abundant in the lepidic pattern, showing nuclear staining. The strongest differences in protein expression between the two patterns using the H-Score was shown for *ITPKA* (p = 0.02) and *angiogenin* (p = 0.113, not significant). Cytoplasmic and nuclear expression of *PFKP* was present in both patterns, slightly more in the solid architecture (not significant). The cytoplasmic expression of *ERRFI1* remained below the 50 point H-score level for both patterns, and was slightly higher expressed in the solid architecture (Fig 1).

## Discussion

For a long time, the nature of adenocarcinomas was histologically and clinically seen as a monolithic block. Clinical research within the last decade has led to a precise definition of subtypes considering growth patterns and molecular characteristics which are associated with diverse clinical outcomes [1,5,13]. However, histopathological growth patterns and prognostic diversity did not necessarily match with molecular subtypes defined by transcriptional profiles, CpG island methylation or oncogene mutations [13]. So far, biomarkers and molecular targets specific for tumor growth patterns have been sporadic. For example, an association was reported between higher expression of TTF-1 and the lepidic pattern [25]. In the solid pattern, C4.4A was reported as a surrogate marker for a poor outcome [26]. The objective of this project was to broaden our knowledge of the different molecular structures between distinct patterns of lung adenocarcinoma. Furthermore, this may help to establish novel biomarkers and target sites that are valuable for future individualised treatment strategies in human pulmonary adenocarcinoma. To our knowledge, we present the first screening study to identify growth pattern-specific gene signatures. Our approach included laser-captured microdissection to separate specific growth patterns in tissue sections of lung adenocarcinoma. Subsequent microarray analyses depicted transcriptome differences between the solid pattern and other growth patterns. A gene classifier could be adopted to assign all 48 gene expression profiles to previously defined molecular subtypes linked to lung adenocarcinomas. For example, the majority of solid specimens matched with the PI subtype. This association was also described in a comprehensive study including 230 adenocarcinomas [13]. Here, upregulated genes in the solid pattern were found to be more frequently aberrant in the PI subtype. Moreover, we suggest molecular similarities between the acinar pattern and the PP subtype as well as the lepidic pattern and the TRU subtype. Of note, both these tumor subgroups have been independently found to be prognostically favourable [5,12].

Microarray profiles revealed a clear separation between the solid and lepidic growth patterns upon tissue microdissection. Further downstream analyses of the gene signature suggested oncogenic targets, pathways and gene ontologies in both growth patterns. Genes upregulated in the worse prognostic solid architecture were associated with cell proliferation, cell cycle activation, inhibition of apoptosis and cell motility. Our signature did not show a significant overlap with existing lung adenocarcinoma microarray data. The poor accordance with other signatures is likely reasonable since microdissected growth patterns are not readily comparable with unselected tumor tissues.

Four candidate genes were selected for IHC protein analyses according to their expression differences between the solid and the lepidic pattern. The IHC results indicated a higher abundance of *ITPKA* in the solid pattern of pulmonary adenocarcinoma. Up-regulation of *ITPKA* in tumor cell lines with low endogenous *ITPKA* expression increases migration [27]. *ITPKA* is preferentially expressed in cell lines derived from metastases of small cell lung cancer and squamous lung carcinoma, whereas pulmonary adenocarcinoma shows a high expression frequency of *ITPKA* in primary tumor cells. The expression of *ITPKA* in adenocarcinoma might increase the invasive potential of cancer cells. Furthermore, this enzyme is considered a potential target for anti-metastatic therapy and small molecule inhibitors [28]. Our results suggest *ITPKA* as a potential target for the development of targeted therapies, particular for predominant solid lung adenocarcinoma.

*Angiogenin* was shown to be up-regulated in the lepidic pattern on the RNA and protein levels. *Angiogenin* is known as a pro-angiogenic growth factor that is up-regulated in several types of cancer. Nuclear expression of *angiogenin* has been shown in about two thirds of lung adenocarcinomas, and target inhibition impairs xenograft tumor proliferation and angiogenesis [29]. A p53 interacting function of *angiogenin* in anti-apoptosis and survival of cancer cells suggests that targeting *angiogenin* could be an effective therapy for several cancers [30]. Our data indicate that *angiogenin* might be of importance in the lepidic architecture, nevertheless our findings were not statistically significant. Upregulation of *PFKP* and *ERRFI1* in the solid architecture could not be confirmed on the protein level. Possible explanations are the discordance between RNA and protein, or additional tumor characteristics as confounding factors. For example, it has been shown that *ERRFI1/MIG6* expression is associated with EMT and resistance to *EGFR* inhibitors in lung cancer xenografts [31].

Overall, this study presents distinct transcriptomic differences between lung adenocarcinoma growth patterns, which could be validated for the solid-expressed *ITPKA* and the lepidic-expressed *angiogenin* proteins. As a limitation, these gene signatures and putative targets could only indirectly be linked to prognosis via previously defined prognostic adenocarcinoma subgroups. Our screening and validation cohort was designed for pattern-specific expression analysis, but not adequate to include clinical follow-up data into statistical analyses. Further studies in larger cohorts of IASLC/ATS/ERS classified adenocarcinomas are needed to better understand the associations between molecular heterogeneity and clinical outcome in pulmonary adenocarcinoma.

## Conclusion

To our knowledge, this study is the first to identify growth pattern-specific gene signatures in pulmonary adenocarcinoma. With tumor profiling at the molecular level the study provides valuable new information about pulmonary adenocarcinoma and allows a better assessment of the five adenocarcinoma subgroups. As this study at hand is only a modest contribution to basic cancer research further validation of individual biomarkers and target sites in distinct histological patterns are strictliy necessary to pave the way toward novel approaches in pathological diagnostics and personalised therapy in the future.

## Supporting information

S1 Fig RIN (RNA integrity number) values of 48 samples. Illumina chip raw data quartiles and RIN values were combined for ordered samples LC1-LC48. Left axis defines log2 expression value quartiles for each chip, numbering on the right assign RIN value for each RNA sample used for the chip.
(TIF)

S2 Fig Heatmap showing SVM predictions for the expression subtype for all 48 specimens in this study. The color code indicates the class probability for each of the three expression subtypes, thus visualizing the level of confidence of the prediction. Notably, the SVM model was trained on the data by Wilkerson et al. based on the overlap of their reported signature and our chip (260 genes).
(TIF)

S3 Fig Multi-dimensional scaling plot of joint expression data from Wilkerson et al. (2012) and this study. Samples from Wilkerson et al. (2012) are colored according to the expression subtype (PI (red), PP (green), TRU (black)). Samples from this study are labeled with a number, linking to S1 Table (“LC_” is omitted from the text labels in this plot to save space).
(TIF)

S4 Fig Comparison of the transcriptomic activity between the solid and lepidic patterns. Hierarchical clustering (Ward’s method) of differentially expressed genes between the solid and lepidic growth patterns using Pearson correlation distance.
(TIF)

S1 Table Differentially expressed genes between all tumor pattern comparisons. S1 Table shows LIMMA analyses and the number of differentially expressed genes (FDR 5%, fold change >1.5 or < 0.66) between all tumor pattern comparisons.
(PDF)

S2 Table Molecular subtype assignment. S2 Table shows molecular subtype assignment using the reported nearest centroid subtype predictor overlap (Wilkerson PlosOne, 2012)
(PDF)

S3 Table Differentially expressed non-redundant genes between solid and lepidic architecture specimens. LIMMA analysis revealed 815 differentially expressed non-redundant genes (FDR 5%, fold change > 1.5 or < 0.66) between solid and lepidic architecture specimens. The gene list is ordered according to a decreasing fold change.
(PDF)

S4 Table Comparison of the 50 highest upregulated genes in solid or lepidic growth pattern with published gene signatures using GeneSigDB. S4 Table shows the comparison of the 50 highest upregulated genes in solid or lepidic growth pattern with published gene signatures using GeneSigDB. Table view was restricted to studies with a minimum of 20% overlap (10 genes).
(PDF)

S5 Table Comparison of known lung adenocarcinoma oncogenes and tumor suppressor genes between different growth patterns. Known lung adenocarcinoma oncogenes and tumor suppressor genes were shown with MEAN and SD expression values in all growth patterns. T-test between different growth patterns were mostly not significant (p-value > 0.05, not adjusted). Only one group comparison indicated higher expression of MET and MAP2K1 (MEK1) in solid compared to lepidic growth pattern (p-value 0.04).
(PDF)
