# Supplementary figures and images for: Molecular signatures in IASLC/ATS/ERS classified growth patterns of lung adenocarcinoma
Source: PLoS One. 2018 Oct 23;13(10):e0206132. doi: 10.1371/journal.pone.0206132 (PMC6198952; doi:10.1371/journal.pone.0206132)

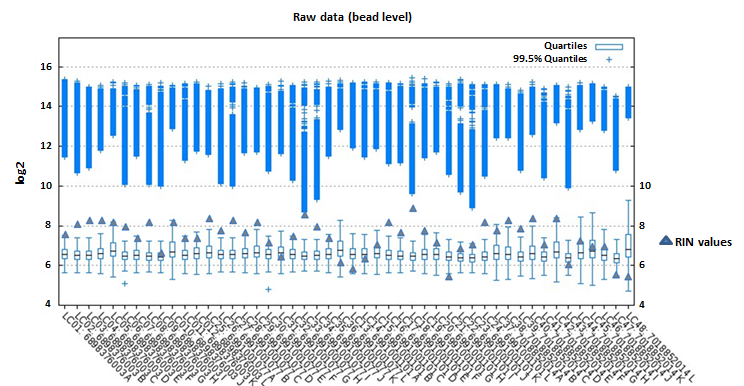

Supplement: S1 Fig — Illumina chip raw data quartiles and RIN values were combined for ordered samples LC1-LC48. Left axis defines log2 expression value quartiles for each chip, numbering on the right assign RIN value for each RNA sample used for the chip. (TIF) [file pone.0206132.s001.tif]

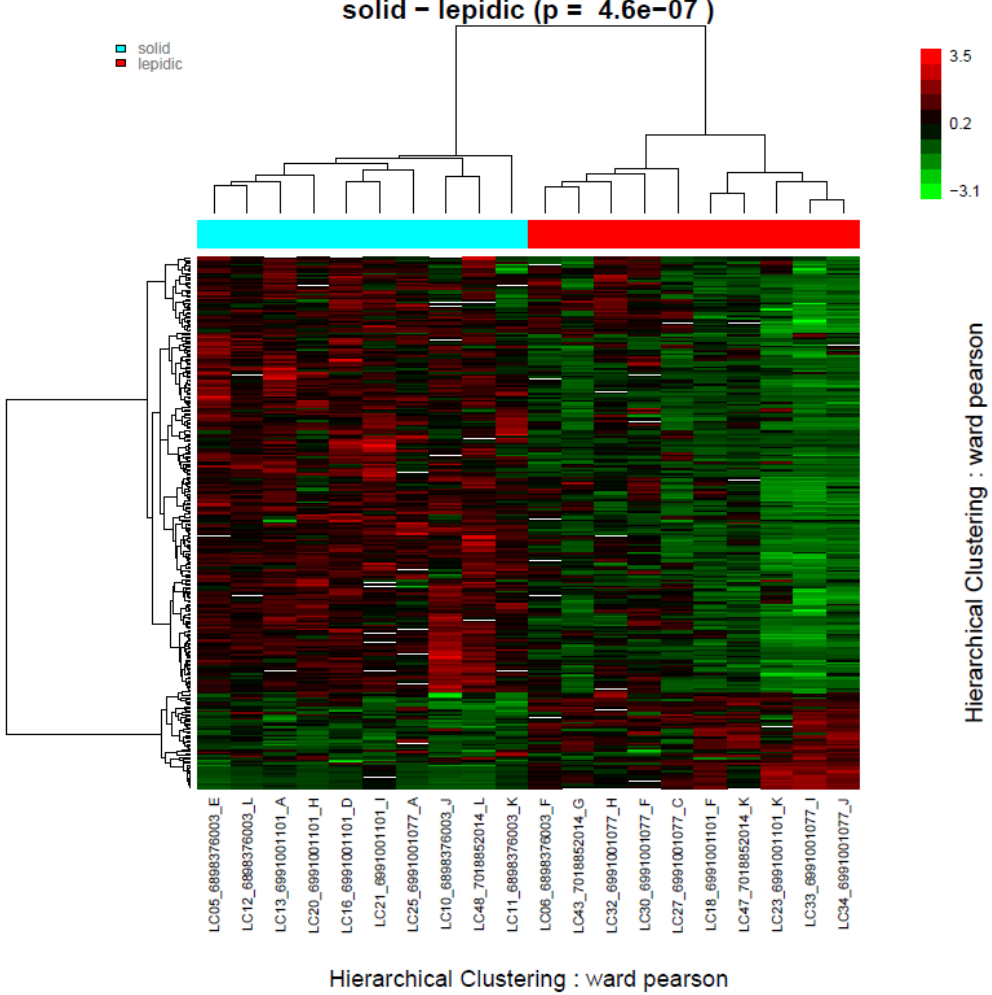

Supplement: S4 Fig — Hierarchical clustering (Ward’s method) of differentially expressed genes between the solid and lepidic growth patterns using Pearson correlation distance. (TIF) [file pone.0206132.s004.tif]
