# Supplementary material for: Molecular signatures in IASLC/ATS/ERS classified growth patterns of lung adenocarcinoma
Source: PLoS One. 2018 Oct 23;13(10):e0206132. doi: 10.1371/journal.pone.0206132 (PMC6198952; doi:10.1371/journal.pone.0206132)
Supplement: S1 Table — shows LIMMA analyses and the number of differentially expressed genes (FDR 5%, fold change >1.5 or < 0.66) between all tumor pattern comparisons. (PDF) [file pone.0206132.s005.pdf]

	solid	lepidic	papillary	micropapillary	acinar
solid	x	815	1321	527	965
lepidic	x	x	0	0	0
papillary	x	x	x	100	4
micropapillary	x	x	x	x	0
acinar	x	x	x	x	x

Supplementary Table 1: LIMMA analyses and the number of differentially expressed genes (FDR 5%, fold change >1.5 or < 0.66) between all tumor pattern comparisons
