# Supplementary material for: Molecular signatures in IASLC/ATS/ERS classified growth patterns of lung adenocarcinoma
Source: PLoS One. 2018 Oct 23;13(10):e0206132. doi: 10.1371/journal.pone.0206132 (PMC6198952; doi:10.1371/journal.pone.0206132)
Supplement: S2 Table — shows molecular subtype assignment using the reported nearest centroid subtype predictor overlap (Wilkerson PlosOne, 2012) (PDF) [file pone.0206132.s006.pdf]

Sample Name	Pattern	Molecular Subtype	Sample Name	Pattern	Molecular Subtype
LC05	solid	proximal-inflammatory	LC02	acinar	proximal-proliferative
LC10	solid	proximal-inflammatory	LC03	acinar	proximal-proliferative
LC11	solid	proximal-inflammatory	LC07	acinar	proximal-proliferative
LC12	solid	proximal-inflammatory	LC15	acinar	proximal-proliferative
LC13	solid	proximal-inflammatory	LC19	acinar	proximal-proliferative
LC16	solid	proximal-inflammatory	LC26	acinar	proximal-proliferative
LC20	solid	proximal-inflammatory	LC36	acinar	proximal-proliferative
LC21	solid	proximal-inflammatory	LC25	solid	terminal respiratory unit
LC48	solid	proximal-inflammatory	LC28	papillary	terminal respiratory unit
LC31	micropapillary	proximal-inflammatory	LC40	papillary	terminal respiratory unit
LC45	micropapillary	proximal-inflammatory	LC44	papillary	terminal respiratory unit
LC04	acinar	proximal-inflammatory	LC08	micropapillary	terminal respiratory unit
LC09	acinar	proximal-inflammatory	LC14	micropapillary	terminal respiratory unit
LC17	papillary	proximal-proliferative	LC29	micropapillary	terminal respiratory unit
LC22	papillary	proximal-proliferative	LC37	micropapillary	terminal respiratory unit
LC24	papillary	proximal-proliferative	LC41	micropapillary	terminal respiratory unit
LC38	papillary	proximal-proliferative	LC06	lepidic	terminal respiratory unit
LC39	papillary	proximal-proliferative	LC18	lepidic	terminal respiratory unit
LC46	papillary	proximal-proliferative	LC32	lepidic	terminal respiratory unit
LC35	micropapillary	proximal-proliferative	LC33	lepidic	terminal respiratory unit
LC42	micropapillary	proximal-proliferative	LC34	lepidic	terminal respiratory unit
LC23	lepidic	proximal-proliferative	LC43	lepidic	terminal respiratory unit
LC27	lepidic	proximal-proliferative	LC47	lepidic	terminal respiratory unit
LC30	lepidic	proximal-proliferative	LC01	acinar	terminal respiratory unit

Supplementary Table 2: Molecular subtype assignment using the reported nearest centroid subtype predictor overlap (Wilkerson PlosOne, 2012)
