# Supplementary material for: Molecular signatures in IASLC/ATS/ERS classified growth patterns of lung adenocarcinoma
Source: PLoS One. 2018 Oct 23;13(10):e0206132. doi: 10.1371/journal.pone.0206132 (PMC6198952; doi:10.1371/journal.pone.0206132)
Supplement: S3 Table — LIMMA analysis revealed 815 differentially expressed non-redundant genes (FDR 5%, fold change > 1.5 or < 0.66) between solid and lepidic architecture specimens. The gene list is ordered according to a decreasing fold change. (PDF) [file pone.0206132.s007.pdf]

ProbeID	Search_Key	ILMN_Gene	Entrez_Gene_ID	Symbol	AveExpr	t	P.Value	adj.P.Val	B	Fold change
2360452	NM_002627.3	PFKP	5214	PFKP	7.197208	-4.320945	8.23E-05	0.004161	1.417969	5.72957622
7100639	NM_018948.2	ERRFI1	54206	ERRFI1	8.000337	-5.2502	3.79E-06	0.000932	4.272147	5.670647848
1110373	NM_032413.2	C15ORF48	84419	C15orf48	7.42657	-3.446173	0.001226	0.016068	-1.056896	5.463789819
110719	NM_004207.2	SLC16A3	9123	SLC16A3	8.614555	-4.803648	1.7E-05	0.001953	2.880016	5.455693622
1430735	NM_005863.2	NET1	10276	NET1	7.75253	-4.878478	1.32E-05	0.001682	3.111047	5.420697838
6590132	NM_001013398.1	IGFBP3	3486	IGFBP3	7.598202	-3.177017	0.002659	0.025178	-1.756943	5.064909841
6200091	NM_001097597.1	XAGE1C	653048	XAGE1C	7.273354	-2.844698	0.006616	0.043509	-2.571094	4.94425506
5340246	NM_001312.2	CRIP2	1397	CRIP2	7.152674	-3.791184	0.000435	0.009052	-0.113486	4.666023179
3460070	NM_000582.2	SPP1	6696	SPP1	7.20707	-3.06018	0.003686	0.030541	-2.049854	4.660364315
6510377	NM_016639.1	TNFRSF12A	51330	TNFRSF12A	7.55157	-3.847543	0.000366	0.008391	0.04502	4.546814111
2100196	NM_005101.1	ISG15	9636	ISG15	8.568666	-3.214764	0.00239	0.023904	-1.660844	4.475775949
990288	NM_201555.1	FHL2	2274	FHL2	7.631024	-2.889312	0.005872	0.040408	-2.465275	4.211751527
4640086	NM_033260.2	FOXQ1	94234	FOXQ1	7.685115	-2.789078	0.007666	0.047676	-2.701428	4.209691855
6560546	NM_002220.1	ITPKA	3706	ITPKA	6.748978	-3.118381	0.003135	0.027948	-1.904813	4.133625891
3130615	NM_013232.2	PDCD6	10016	PDCD6	8.047418	-4.471886	5.05E-05	0.003323	1.869328	4.087051387
20546	NM_001306.2	CLDN3	1365	CLDN3	7.593439	-3.40235	0.001393	0.017342	-1.173157	4.052616054
1710189	NM_022450.2	RHBDF1	64285	RHBDF1	7.224611	-4.378997	6.83E-05	0.003695	1.59087	3.787250534
3850433	NM_005346.3	HSPA1B	3304	HSPA1B	8.225834	-4.763561	1.94E-05	0.002025	2.756678	3.700239063
5690519	NM_006227.2	PLTP	5360	PLTP	7.643935	-2.812598	0.007205	0.045932	-2.646533	3.650551395
4890270	NM_002346.1	LY6E	4061	LY6E	10.41805	-3.809074	0.000412	0.008851	-0.063298	3.573258374
4590370	NM_006516.1	SLC2A1	6513	SLC2A1	7.018844	-3.025708	0.004054	0.032525	-2.134927	3.5669732
290114	NM_006824.1	EBNA1BP2	10969	EBNA1BP2	7.739781	-6.873397	1.42E-08	5.41E-05	9.46216	3.427021357
1300113	NM_004360.2	CDH1	999	CDH1	8.127848	-3.533372	0.000947	0.013933	-0.823067	3.381425284
6840372	NM_001013398.1	IGFBP3	3486	IGFBP3	6.929849	-2.858156	0.006383	0.042848	-2.539293	3.354742707
160598	NM_006644.2	HSPH1	10808	HSPH1	7.520795	-4.592304	3.41E-05	0.002738	2.233408	3.315140316
3170594	NM_052886.1	MAL2	114569	MAL2	7.12674	-3.908516	0.000303	0.007584	0.217783	3.300859506
4050711	NM_016027.1	LACTB2	51110	LACTB2	6.690021	-4.078772	0.000178	0.005909	0.706863	3.297895387
6100022	NM_003517.2	HIST2H2AC	8338	HIST2H2AC	7.419876	-2.92488	0.005336	0.038379	-2.380112	3.278172444
6620392	NM_002304.1	LFNG	3955	LFNG	7.25546	-2.849876	0.006526	0.043271	-2.558871	3.219901946
110468	NM_080388.1	S100A16	140576	S100A16	7.569036	-3.096991	0.003328	0.02891	-1.958322	3.208434883
2070368	NM_006088.5	TUBB2C	10383	TUBB2C	7.28018	-4.192305	0.000124	0.004966	1.038111	3.187119219
160170	NM_182471.1	PKM2	5315	PKM2	8.43005	-4.135102	0.000149	0.005334	0.870726	3.178871205
6940360	NM_001105.2	ACVR1	90	ACVR1	7.277116	-6.299909	1.03E-07	0.000131	7.622026	3.178639506
5820129	NM_002568.3	PABPC1	26986	PABPC1	9.500983	-4.141448	0.000146	0.005334	0.889246	3.162121334
1690553	NM_002354.2	EPCAM	4072	EPCAM	9.049272	-3.832061	0.000384	0.008619	0.001361	3.161762364
6380717	NM_005345.4	HSPA1A	3303	HSPA1A	7.519371	-3.85224	0.000361	0.008346	0.058281	3.141293383
20044	XM_932346.1	LOC644743	644743	LOC644743	8.021131	-3.151479	0.002858	0.026272	-1.821557	3.091144161
4560717	NM_001012334.1	MDK	4192	MDK	9.580069	-2.925369	0.005329	0.038379	-2.378935	3.062666577
3310091	NM_018643.2	TREM1	54210	TREM1	6.026612	-3.302033	0.001863	0.020661	-1.436017	3.056577034
3170273	NM_013451.2	FER1L3	26509	FER1L3	7.022706	-3.812375	0.000408	0.008851	-0.054024	3.053752732
5220767	NM_001457.1	FLNB	2317	FLNB	7.0068	-3.678639	0.000613	0.010609	-0.426467	3.048802691
5270730	NM_001101.2	ACTB	60	ACTB	7.621693	-3.257625	0.002116	0.022257	-1.55088	3.047760367
6580270	NM_199187.1	LOC646723	646723	LOC646723	9.077642	-4.158466	0.000138	0.005264	0.938975	3.047260221
3450093	NM_017755.4	NSUN2	54888	NSUN2	7.556019	-4.217747	0.000115	0.004766	1.112866	3.032681224
3780092	NM_014452.3	TNFRSF21	27242	TNFRSF21	8.83718	-3.206991	0.002443	0.024089	-1.68069	3.022792118
2140528	NM_024531.3	GPR172A	79581	GPR172A	8.219333	-4.157895	0.000139	0.005264	0.937304	3.011578791
1070133	NM_012129.2	CLDN12	9069	CLDN12	7.10755	-4.041584	0.0002	0.006067	0.59923	2.970284934
5870474	NM_014899.2	RHOBTB3	22836	RHOBTB3	7.168091	-2.91085	0.005542	0.039098	-2.413789	2.942980499
3800161	NM_005556.3	KRT7	3855	KRT7	7.633092	-2.807601	0.0073	0.046308	-2.658222	2.937722754
2100678	NM_052951.2	DNTTIP1	116092	DNTTIP1	7.011528	-4.027251	0.000209	0.006191	0.557863	2.909162323
1980553	NM_003815.3	ADAM15	8751	ADAM15	7.179338	-4.39404	6.5E-05	0.003596	1.635818	2.90032376
2480039	NM_015878.4	AZIN1	51582	AZIN1	7.865542	-4.56788	3.69E-05	0.002865	2.159293	2.8827478
4180343	NM_003406.2	YWHAZ	7534	YWHAZ	9.47424	-4.906591	1.2E-05	0.001612	3.198103	2.855110448
1410161	NM_001007075.1	KLHL5	51088	KLHL5	6.700395	-3.402295	0.001393	0.017342	-1.173303	2.849388932
4120193	NM_144707.1	PROM2	150696	PROM2	7.432221	-2.883998	0.005957	0.040768	-2.477937	2.811482332
7000017	NM_016289.2	CAB39	51719	CAB39	7.764373	-4.503132	4.56E-05	0.003137	1.963475	2.809962098
520196	NM_013285.1	GNL2	29889	GNL2	7.337155	-5.314892	3.04E-06	0.000829	4.476151	2.783353054
1660435	NM_015201.3	BOP1	23246	BOP1	7.206142	-3.295563	0.001898	0.020867	-1.452811	2.776552445
1340075	NM_004281.3	BAG3	9531	BAG3	6.508377	-4.887597	1.28E-05	0.001659	3.139272	2.762809595
4780671	NM_014584.1	ERO1L	30001	ERO1L	7.092292	-3.319202	0.001773	0.020284	-1.391359	2.760445213
3800309	NM_022170.1	EIF4H	7458	EIF4H	7.754197	-4.43567	5.68E-05	0.003472	1.760507	2.746744273
6550092	XR_016557.2	LOC391075	391075	LOC391075	8.591499	-4.028837	0.000208	0.006187	0.562436	2.742989857
3310025	NM_152398.2	OCIAD2	132299	OCIAD2	7.45479	-3.138415	0.002964	0.026993	-1.854485	2.738696147
6110026	XM_001726959.1	KRT18P13	392371	KRT18P13	8.071323	-3.302721	0.001859	0.02065	-1.43423	2.730774103
5670368	NM_014437.3	SLC39A1	27173	SLC39A1	8.17481	-4.618432	3.13E-05	0.002713	2.312842	2.708716101

5080451	NM_002790.2	PSMA5	5686	PSMA5	9.007073	-5.405503	2.23E-06	0.000721	4.762685	2.683300016
6840246	NM_000308.1	CTSA	5476	CTSA	6.89278	-3.780742	0.000449	0.009123	-0.142727	2.682000074
2340072	NM_022750.2	PARP12	64761	PARP12	6.651693	-4.162742	0.000137	0.005264	0.951484	2.680415953
4050040	NM_006086.2	TUBB3	10381	TUBB3	6.300809	-3.356821	0.00159	0.018955	-1.29303	2.649540056
7570215	NM_003909.2	CPNE3	8895	CPNE3	8.1807	-3.808986	0.000412	0.008851	-0.063544	2.629036632
3520156	NM_006585.2	CCT8	10694	CCT8	7.309488	-3.932521	0.000282	0.007239	0.286156	2.618670713
6980100	NM_006295.1	VARS	7407	VARS	7.418752	-3.915875	0.000296	0.00749	0.238723	2.617540681
3180470	NM_004168.1	SDHA	6389	SDHA	7.269293	-3.271915	0.002031	0.021885	-1.514021	2.611871275
7320041	NM_015892.2	CHST15	51363	CHST15	6.850728	-3.245264	0.002191	0.022672	-1.582685	2.605564795
3420632	NM_004159.4	PSMB8	5696	PSMB8	7.102827	-3.484689	0.001094	0.015269	-0.954018	2.601564651
2650114	XM_944693.1	ITGB5	3693	ITGB5	7.693867	-3.499463	0.001048	0.014886	-0.914385	2.59945496
990193	NM_005850.3	SF3B4	10262	SF3B4	8.066268	-4.096338	0.000168	0.005689	0.757858	2.576690278
150681	NM_017953.2	ZNHIT6	54680	ZNHIT6	6.07462	-5.043531	7.61E-06	0.001172	3.624043	2.561200431
4280202	XR_018212.1	LOC399748	399748	LOC399748	7.062009	-3.737448	0.000513	0.009718	-0.263524	2.533552328
3370092	NM_013233.1	STK39	27347	STK39	7.437588	-3.331261	0.001712	0.019889	-1.35991	2.529007207
5690437	NM_004515.2	ILF2	3608	ILF2	8.772857	-4.24065	0.000107	0.004633	1.180321	2.518603457
3180672	NM_004862.2	LITAF	9516	LITAF	7.873327	-3.034058	0.003961	0.032023	-2.114377	2.514764431
2030093	NM_182470.1	PKM2	5315	PKM2	6.203444	-4.966057	9.87E-06	0.001395	3.382695	2.514489567
4390121	NM_007002.2	ADRM1	11047	ADRM1	7.282115	-4.614643	3.17E-05	0.002716	2.301311	2.512963442
6650736	NM_130395.1	WRNIP1	56897	WRNIP1	7.545344	-3.956059	0.000262	0.007011	0.353388	2.508395021
430307	NM_012428.1	NPTN	27020	NPTN	6.67409	-4.521548	4.3E-05	0.003036	2.019072	2.493843531
1570672	NM_005186.2	CAPN1	823	CAPN1	7.485868	-3.539826	0.000929	0.013799	-0.805631	2.493801803
5290132	NM_030582.2	COL18A1	80781	COL18A1	6.322966	-3.736475	0.000514	0.009718	-0.266231	2.486781504
4210239	NM_014078.4	MRPL13	28998	MRPL13	7.793063	-3.500215	0.001045	0.01488	-0.912365	2.466494574
4830100	NM_007355.2	HSP90AB1	3326	HSP90AB1	8.705119	-4.395504	6.47E-05	0.003596	1.640196	2.46594442
1240482	NM_006014.2	LAGE3	8270	LAGE3	7.145271	-3.262747	0.002085	0.022189	-1.537679	2.465354357
7380221	NM_015535.1	SPATS2L	26010	SPATS2L	6.794942	-3.394436	0.001426	0.017604	-1.194061	2.463032683
3290689	NM_016039.1	C14ORF166	51637	C14orf166	7.284047	-4.339797	7.75E-05	0.004072	1.474021	2.4623692
2470097	NM_037370.1	CCNDBP1	23582	CCNDBP1	7.359132	-3.967982	0.000252	0.006908	0.387515	2.436916218
2350053	NM_002056.1	GFPT1	2673	GFPT1	7.583142	-3.012971	0.004198	0.033196	-2.166201	2.430815755
4210309	NM_016040.3	TMED5	50999	TMED5	7.626111	-4.04437	0.000198	0.006067	0.607279	2.427927321
3440224	NM_024640.3	YRDC	79693	YRDC	6.306455	-5.466013	1.82E-06	0.00066	4.954507	2.422379773
2190189	NM_015934.3	NOP58	51602	NOP58	7.345117	-5.101993	6.25E-06	0.001163	3.806775	2.418394174
3800392	NM_001009184.1	GRINA	2907	GRINA	6.719758	-3.405311	0.001381	0.017294	-1.165329	2.411127969
2630463	NM_002810.2	PSMD4	5710	PSMD4	7.615298	-3.702481	0.00057	0.010218	-0.36057	2.410193822
870537	NR_000030.1	LOC134997	134997	LOC134997	8.68809	-3.055214	0.003737	0.030861	-2.062147	2.408249882
6330270	NM_001448.2	GPC4	2239	GPC4	6.348903	-2.819285	0.007078	0.045504	-2.630865	2.398086206
1690500	NM_001020658.1	PUM1	9698	PUM1	7.835133	-4.955967	1.02E-05	0.001416	3.351333	2.39731166
6770619	NM_006708.1	GLO1	2739	GLO1	7.602303	-3.388838	0.001449	0.017835	-1.208831	2.390014788
2070088	NM_002127.3	HLA-G	3135	HLA-G	6.075413	-3.315499	0.001792	0.020361	-1.401002	2.385242558
5080280	NM_020210.2	SEMA4B	10509	SEMA4B	6.839852	-2.919665	0.005412	0.038777	-2.392642	2.37888573
3460121	NM_014765.1	TOMM20	9804	TOMM20	6.414011	-3.582604	0.000818	0.012743	-0.689622	2.377425088
4070575	NM_002354.1	TACSTD1	4072	TACSTD1	10.10011	-2.99747	0.00438	0.034003	-2.204147	2.376597497
1470184	NM_001009566.1	CLSTN1	22883	CLSTN1	8.204731	-3.259264	0.002106	0.022257	-1.546656	2.376054397
6100288	NM_004261.3	15-Sep	9403	SEP15	8.270363	-4.109964	0.000161	0.005573	0.797479	2.372829602
940754	NM_138391.3	TMEM183A	92703	TMEM183A	7.267253	-3.470343	0.001141	0.015499	-0.992412	2.372811404
2060152	NM_019051.1	MRPL50	54534	MRPL50	6.657359	-4.163459	0.000136	0.005264	0.953582	2.371446672
4050639	NM_006452.2	PAICS	10606	PAICS	6.830671	-3.413656	0.001348	0.017058	-1.143244	2.368755203
2490240	NM_130435.2	PTPRE	5791	PTPRE	6.504488	-3.311896	0.001811	0.020398	-1.41038	2.349958699
6250553	NM_032039.1	ITFG3	83986	ITFG3	7.15072	-3.124735	0.00308	0.027586	-1.888874	2.33835591
4040564	NM_001025243.1	IRAK1	3654	IRAK1	8.003518	-3.723003	0.000536	0.009946	-0.30367	2.334574476
2650370	NM_000449.3	RFX5	5993	RFX5	6.650558	-3.614806	0.000743	0.011962	-0.601797	2.332688284
5490470	NM_002463.1	MX2	4600	MX2	6.542368	-3.597172	0.000783	0.012428	-0.649942	2.329774273
270152	NM_003486.5	SLC7A5	8140	SLC7A5	6.284205	-3.470553	0.001141	0.015499	-0.991851	2.329089697
5860315	NM_058246.3	DNAJB6	10049	DNAJB6	6.267188	-5.048678	7.48E-06	0.001172	3.640111	2.327466927
3850010	NM_001889.2	CRYZ	1429	CRYZ	8.284279	-2.824817	0.006975	0.045146	-2.617885	2.32672769
6220300	NM_004965.6	HMGN1	3150	HMGN1	9.020289	-4.297385	8.88E-05	0.004344	1.348054	2.32672127
5270689	NM_006353.2	HMGN4	10473	HMGN4	7.035545	-4.27382	9.58E-05	0.004485	1.278279	2.323407678
160731	NM_016479.3	SHISA5	51246	SHISA5	8.026264	-3.362072	0.001566	0.018882	-1.279253	2.318100828
2320093	NM_032906.2	PIGY	84992	PIGY	8.012505	-3.515855	0.000998	0.01445	-0.870303	2.313155537
2490411	NM_002213.3	ITGB5	3693	ITGB5	7.031391	-3.300763	0.00187	0.020707	-1.439314	2.308726418
4780040	NM_032477.1	MRPL41	64975	MRPL41	7.143776	-3.065423	0.003633	0.030384	-2.036859	2.298716728
6200369	NM_206876.1	PPP1CB	5500	PPP1CB	6.643264	-4.540012	4.05E-05	0.002912	2.074892	2.297262117
2450139	NM_005614.2	RHEB	6009	RHEB	8.474833	-3.378863	0.001492	0.018156	-1.235114	2.297169133
2120451	NM_172358.1	CD46	4179	CD46	7.855854	-3.320713	0.001765	0.020226	-1.387422	2.292449743
4880370	NM_021991.1	JUP	3728	JUP	8.707226	-3.183879	0.002608	0.024943	-1.739525	2.287612651

2690047	NM_015161.1	ARL6IP1	23204	ARL6IP1	7.935997	-3.086478	0.003427	0.029269	-1.984535	2.286716655
1470706	NM_016647.1	C8ORF55	51337	C8orf55	7.74385	-2.805852	0.007334	0.046446	-2.662309	2.284903563
3850414	NM_014366.4	GNL3	26354	GNL3	6.846098	-3.811035	0.00041	0.008851	-0.057789	2.283838693
5310170	NM_020441.2	CORO1B	57175	CORO1B	7.444266	-3.907619	0.000304	0.007584	0.21523	2.280976582
3940670	NM_005219.3	DIAPH1	1729	DIAPH1	6.384774	-4.250799	0.000103	0.004552	1.210259	2.280905269
5050537	NM_017570.1	OPLAH	26873	OPLAH	6.503563	-2.986276	0.004516	0.034707	-2.231468	2.268670921
6290546	NM_033224.3	PURB	5814	PURB	6.562284	-4.770399	1.89E-05	0.002007	2.777695	2.265996593
7650484	NM_005998.3	CCT3	7203	CCT3	6.666354	-3.629954	0.00071	0.011677	-0.560338	2.263212453
6290603	NM_003751.2	EIF3B	8662	EIF3B	7.411306	-3.250792	0.002157	0.02255	-1.568469	2.261751248
620711	NM_006819.1	STIP1	10963	STIP1	7.580041	-4.19328	0.000124	0.004966	1.04097	2.255881407
2850112	NM_016172.2	UBAC1	10422	UBAC1	6.566531	-5.044106	7.59E-06	0.001172	3.625839	2.252002153
2970730	NM_001020820.1	MYADM	91663	MYADM	7.310605	-2.885851	0.005927	0.040675	-2.473525	2.251469326
460519	NM_005833.2	RABEPK	10244	RABEPK	6.902447	-4.040079	0.000201	0.006067	0.594882	2.246324341
7200600	NM_018206.3	VPS35	55737	VPS35	7.871901	-2.893158	0.005812	0.040174	-2.456099	2.245464948
5130164	NM_002890.1	RASA1	5921	RASA1	6.936432	-3.221103	0.002347	0.02357	-1.644636	2.240585639
6770601	XM_001126471.1	LOC730278	730278	LOC730278	8.381978	-2.853618	0.006461	0.043132	-2.550026	2.238802397
4010347	NM_006837.2	COPS5	10987	COPS5	7.952029	-2.967568	0.004753	0.035732	-2.276978	2.238293702
5420575	NM_001012661.1	SLC3A2	6520	SLC3A2	6.955367	-3.304849	0.001848	0.020614	-1.428701	2.230016329
5090088	NM_004446.2	EPRS	2058	EPRS	7.911502	-3.568507	0.000853	0.013156	-0.727935	2.22366406
6250053	NM_004339.2	PTTG1IP	754	PTTG1IP	6.584627	-3.512583	0.001008	0.014532	-0.87911	2.222391369
830201	NM_015516.3	TSKU	25987	TSKU	6.596494	-2.846566	0.006583	0.043503	-2.566687	2.220002902
5720364	NM_001079809.1	TMEM183B	653659	TMEM183B	7.285649	-3.211879	0.00241	0.023974	-1.668213	2.216448349
3890551	NM_181555.1	CMTM3	123920	CMTM3	6.756145	-2.769145	0.008078	0.049316	-2.747702	2.210028905
160630	NM_001084.4	PLOD3	8985	PLOD3	7.312177	-2.844061	0.006627	0.043509	-2.572597	2.207537434
1470242	NM_014142.2	NUDT5	11164	NUDT5	6.287992	-3.549659	0.000903	0.013559	-0.779031	2.202740608
3710537	NM_000687.1	AHCY	191	AHCY	7.828018	-3.138834	0.002961	0.026993	-1.853431	2.196906002
3850154	NM_178812.2	MTDH	92140	MTDH	7.3746	-3.092168	0.003373	0.02903	-1.970354	2.187960031
1230044	NM_003143.1	SSBP1	6742	SSBP1	7.4803	-2.950104	0.004984	0.036674	-2.319291	2.18531126
5080373	NM_001307.3	CLDN7	1366	CLDN7	8.877366	-2.809425	0.007265	0.046162	-2.653957	2.18204499
60128	NM_014754.1	PTDSS1	9791	PTDSS1	6.342413	-4.828826	1.56E-05	0.00186	2.957636	2.175799028
630086	NM_199342.1	CCDC23	374969	CCDC23	6.626911	-4.363427	7.18E-05	0.003831	1.544409	2.17139873
6980327	NM_001363.2	DKC1	1736	DKC1	7.612781	-3.101431	0.003287	0.028844	-1.947233	2.171388114
5220035	NM_020127.1	TUFT1	7286	TUFT1	6.640029	-3.481591	0.001104	0.015269	-0.962317	2.170631238
6900750	NM_001527.1	HDAC2	3066	HDAC2	6.941553	-3.202722	0.002473	0.024242	-1.691577	2.17057808
4760255	NM_004309.3	ARHGDIA	396	ARHGDIA	7.178662	-3.235853	0.002251	0.023057	-1.606848	2.167676116
50397	NM_004766.1	COPB2	9276	COPB2	6.566456	-4.338023	7.79E-05	0.004072	1.468742	2.165784247
2710195	NM_152295.3	TARS	6897	TARS	6.406407	-3.865214	0.000347	0.008067	0.094955	2.163405651
6250576	NM_181513.1	MRPL21	219927	MRPL21	6.685384	-4.565837	3.72E-05	0.002865	2.153101	2.159688235
5810373	XR_001087.1	FAM127B	26071	FAM127B	6.382164	-4.132822	0.00015	0.005334	0.864074	2.154518986
4290537	NM_006815.2	TMED2	10959	TMED2	8.218847	-3.149297	0.002875	0.026402	-1.827065	2.151602923
6290241	NM_002354.1	TACSTD1	4072	TACSTD1	7.202199	-2.866038	0.00625	0.042171	-2.52062	2.149830968
6980601	XM_001723016.1	LOC729978	729978	LOC729978	8.204952	-3.213679	0.002397	0.023915	-1.663617	2.148221124
1260520	NM_004837.2	GGPS1	9453	GGPS1	6.499541	-5.424532	2.09E-06	0.000721	4.82297	2.147840012
6560162	NM_181836.3	TMED7	51014	TMED7	7.058921	-3.316339	0.001788	0.02036	-1.398816	2.146553671
2640551	NM_016308.1	CMPK1	51727	CMPK1	7.246553	-3.564366	0.000864	0.013264	-0.739175	2.142961169
7380068	NM_003418.1	CNBP	7555	CNBP	6.908259	-3.400609	0.0014	0.017374	-1.177759	2.142494009
6590253	NM_184041.1	ALDOA	226	ALDOA	9.490928	-3.534239	0.000945	0.013933	-0.820726	2.141647642
3460324	NM_152132.1	PSMA3	5684	PSMA3	7.002197	-3.007196	0.004265	0.033475	-2.180353	2.140424598
2760209	XR_037398.1	LOC100132992	100132992	LOC100132992	6.885419	-3.84836	0.000365	0.008391	0.047326	2.139515602
2060047	NM_004776.2	B4GALT5	9334	B4GALT5	6.491633	-3.373445	0.001515	0.018356	-1.249371	2.13660104
2350368	NM_032194.1	RPF2	84154	RPF2	6.56177	-4.662558	2.71E-05	0.002518	2.447325	2.127212557
1940360	NM_000365.4	TPI1	7167	TPI1	8.553936	-3.068935	0.003598	0.030256	-2.028147	2.124639236
5870482	NM_006618.3	KDM5B	10765	KDM5B	7.108284	-3.068549	0.003601	0.030256	-2.029105	2.123910675
4670592	NM_013451.3	MYOF	26509	MYOF	6.538439	-3.166531	0.002739	0.025742	-1.783515	2.123065064
150767	NM_003860.2	BANF1	8815	BANF1	7.559106	-3.089487	0.003398	0.029175	-1.977037	2.11647764
2680064	NM_032431.2	SYVN1	84447	SYVN1	7.131923	-3.129583	0.003038	0.027472	-1.876697	2.1146654
1570619	NM_018845.1	RAG1AP1	55974	RAG1AP1	6.599823	-3.74129	0.000507	0.009697	-0.252832	2.114625602
6960056	NM_006303.2	AIMP2	7965	AIMP2	6.792273	-3.313077	0.001805	0.020398	-1.407307	2.108661577
7210326	NM_004159.4	PSMB8	5696	PSMB8	7.868994	-2.833889	0.006809	0.044449	-2.596562	2.108179553
6580672	NM_203291.1	RBBP8	5932	RBBP8	6.091002	-5.529572	1.46E-06	0.000657	5.156366	2.106851138
5570524	NM_052940.3	LRRC42	115353	LRRC42	6.252081	-4.754418	2E-05	0.002059	2.728591	2.105672807
5700343	NM_018842.3	BAIAP2L1	55971	BAIAP2L1	6.605381	-3.004291	0.004299	0.033579	-2.187466	2.105431432
3440064	XR_037260.1	LOC729406	729406	LOC729406	7.48265	-2.79309	0.007585	0.04747	-2.692088	2.095173202
4880333	NM_173624.1	FLJ40504	284085	FLJ40504	9.783845	-3.040329	0.003894	0.031676	-2.09892	2.092440071
3060347	NM_004623.2	TTC4	7268	TTC4	6.798954	-4.57627	3.59E-05	0.002856	2.184739	2.092002882
5340021	NM_012316.3	KPNA6	23633	KPNA6	6.324119	-5.049261	7.46E-06	0.001172	3.641932	2.091235289

1260162	NM_001379.1	DNMT1	1786	DNMT1	7.579951	-3.07864	0.003502	0.029693	-2.004039	2.089546334
2630022	NM_006711.2	RNPS1	10921	RNPS1	7.105362	-3.339343	0.001673	0.019557	-1.338794	2.089031871
3930086	NM_017859.2	UCKL1	54963	UCKL1	6.84937	-3.896407	0.000315	0.007726	0.18337	2.086845865
4490142	NM_178564.2	NRBP2	340371	NRBP2	6.355885	-3.591175	0.000798	0.012556	-0.666287	2.083629078
1260341	NM_001560.2	IL13RA1	3597	IL13RA1	7.156278	-3.056663	0.003722	0.030771	-2.058562	2.081553173
1050600	NM_002755.2	MAP2K1	5604	MAP2K1	6.311403	-5.016982	8.32E-06	0.001245	3.541231	2.076193883
1980239	XR_038252.1	LOC649553	649553	LOC649553	6.444634	-3.888066	0.000323	0.007827	0.159693	2.076106384
4230296	NM_002803.2	PSMC2	5701	PSMC2	7.901221	-2.940299	0.005118	0.037196	-2.342975	2.076000089
4590725	NM_015027.2	PDXDC1	23042	PDXDC1	7.92311	-2.903984	0.005645	0.039385	-2.430229	2.074290052
130603	NM_014183.2	DYNLRB1	83658	DYNLRB1	7.63757	-3.22863	0.002298	0.023261	-1.625366	2.073540194
2940451	NM_004251.3	RAB9A	9367	RAB9A	6.777746	-3.144887	0.002911	0.026603	-1.838183	2.07262224
4640626	NM_001040181.1	CLDND1	56650	CLDND1	6.75355	-3.834582	0.000381	0.008578	0.008466	2.069930577
7380193	NM_005719.2	ARPC3	10094	ARPC3	7.845446	-2.79482	0.007551	0.047424	-2.688056	2.068793769
1740494	NM_006114.1	TOMM40	10452	TOMM40	7.289685	-3.51877	0.000989	0.014412	-0.862449	2.068028026
5960097	NM_001126.2	ADSS	159	ADSS	7.675679	-3.558951	0.000878	0.013339	-0.753859	2.066841207
610750	NM_021960.3	MCL1	4170	MCL1	7.029538	-3.744441	0.000502	0.009661	-0.244061	2.055299876
1770520	NM_001916.2	CYC1	1537	CYC1	8.775188	-3.073888	0.003549	0.029954	-2.015849	2.05209977
3360059	NM_032982.2	CASP2	835	CASP2	7.326314	-2.861683	0.006323	0.042514	-2.530941	2.050818072
3190059	NM_006839.1	IMMT	10989	IMMT	6.748961	-4.03141	0.000207	0.006187	0.569859	2.047005752
770142	NM_173834.2	YIPF6	286451	YIPF6	6.575767	-4.285587	9.22E-05	0.004372	1.313102	2.046312877
3180215	NM_033657.1	DAP3	7818	DAP3	7.520322	-3.099684	0.003303	0.028849	-1.951597	2.042641151
3780056	NM_024051.2	GGCT	79017	GGCT	6.591795	-3.015717	0.004166	0.033119	-2.159467	2.041017683
1300072	NR_003273.1	SRP14P1	390284	SRP14P1	7.370039	-2.85184	0.006492	0.043226	-2.55423	2.040650758
3940148	NM_020412.3	CHMP1B	57132	CHMP1B	6.331343	-3.450673	0.001209	0.016031	-1.04491	2.038904632
4900639	NM_024835.2	GGNBP2	79893	GGNBP2	6.681925	-5.690881	8.42E-07	0.000494	5.670174	2.038578982
4920379	XR_038201.1	LOC401076	401076	LOC401076	7.13469	-3.354664	0.0016	0.019021	-1.298687	2.032855279
6370538	NM_017528.2	WBSCR22	114049	WBSCR22	7.100768	-2.773114	0.007995	0.048962	-2.738508	2.030492488
2760427	NM_002863.3	PYGL	5836	PYGL	6.272195	-3.101268	0.003288	0.028844	-1.947642	2.030040956
6020491	NM_003092.3	SNRPB2	6629	SNRPB2	7.868869	-2.818623	0.007091	0.045528	-2.632417	2.023603163
20544	NM_000819.3	GART	2618	GART	6.587611	-3.904809	0.000307	0.007622	0.207243	2.022000941
2480487	NM_005819.4	STX6	10228	STX6	6.3294	-5.140962	5.48E-06	0.00113	3.92885	2.020378632
4070195	NM_170750.1	PSMD10	5716	PSMD10	6.58107	-4.072265	0.000182	0.005979	0.687999	2.019082232
2000577	NM_005828.2	DCAF7	10238	DCAF7	6.863504	-3.716966	0.000546	0.00999	-0.320426	2.011675955
4570441	NM_022168.2	IFIH1	64135	IFIH1	5.943089	-2.891082	0.005845	0.040289	-2.461053	2.010451725
1300674	NM_003824.2	FADD	8772	FADD	6.139998	-4.288055	9.15E-05	0.004372	1.32041	2.009936498
6280458	NM_001706.2	BCL6	604	BCL6	7.001858	-2.977078	0.004631	0.035269	-2.253869	2.006356152
870609	XR_017616.1	LOC341315	341315	LOC341315	6.174929	-2.779354	0.007865	0.048399	-2.724031	2.002118583
7050470	NM_006024.4	TAX1BP1	8887	TAX1BP1	8.587598	-2.905784	0.005618	0.039368	-2.425922	1.999890102
2230603	NM_003664.3	AP3B1	8546	AP3B1	6.807922	-4.145334	0.000144	0.005334	0.900594	1.99811022
2600452	NM_006753.3	SURF6	6838	SURF6	6.448746	-3.61295	0.000747	0.012004	-0.60687	1.994613405
3930035	NM_006759.3	UGP2	7360	UGP2	7.02893	-3.288859	0.001935	0.02115	-1.470189	1.993973299
610762	NM_015959.1	TXNDC14	51075	TXNDC14	6.934556	-3.343714	0.001652	0.019452	-1.327364	1.993147039
2000064	NM_175859.1	CTPS2	56474	CTPS2	6.481472	-3.733771	0.000519	0.009774	-0.27375	1.991835019
6420731	NM_005415.3	SLC20A1	6574	SLC20A1	6.406814	-3.84297	0.000371	0.008434	0.032115	1.991790485
2940079	NM_020189.4	ENY2	56943	ENY2	8.460118	-3.092474	0.00337	0.02903	-1.969592	1.990154261
1580524	NM_203372.1	ACSL3	2181	ACSL3	6.568858	-3.044358	0.003851	0.031426	-2.088977	1.988604645
4260189	NM_170695.2	TGIF1	7050	TGIF1	6.431741	-4.142113	0.000146	0.005334	0.891188	1.978392405
7610152	NM_014847.2	UBAP2L	9898	UBAP2L	6.676519	-3.615534	0.000741	0.011962	-0.599805	1.974495266
60148	NM_001031827.1	BOLA2	552900	BOLA2	8.307262	-2.952945	0.004945	0.036533	-2.31242	1.971260133
7560367	NM_005903.5	SMAD5	4090	SMAD5	7.205158	-2.814118	0.007176	0.045822	-2.642974	1.970849436
1340750	NM_014230.2	SRP68	6730	SRP68	7.118703	-3.709124	0.000559	0.010109	-0.34217	1.970423631
540717	NM_004793.2	LONP1	9361	LONP1	6.613243	-4.0412	0.0002	0.006067	0.598121	1.967838743
5340193	NM_004869.2	VPS4B	9525	VPS4B	6.663957	-3.196775	0.002515	0.024417	-1.706729	1.967172836
130685	NM_018184.2	ARL8B	55207	ARL8B	6.490451	-4.180847	0.000129	0.005123	1.004504	1.966525183
2690390	XR_001115.1	HCG4	54435	HCG4	6.585897	-3.461478	0.001172	0.015686	-1.016094	1.964915243
3140364	NM_002835.2	PTPN12	5782	PTPN12	6.728821	-3.509308	0.001017	0.014622	-0.887923	1.963693338
4540181	XR_036947.1	LOC100130308	100130308	LOC100130308	8.422117	-2.820498	0.007055	0.045473	-2.628021	1.960140041
2850128	NM_002268.3	KPNA4	3840	KPNA4	6.771959	-4.419461	5.99E-05	0.003489	1.711907	1.959578832
5890112	NM_022735.3	ACBD3	64746	ACBD3	7.682259	-3.164457	0.002755	0.025797	-1.788761	1.958287866
870176	NM_006070.4	TFG	10342	TFG	6.714258	-4.425748	5.87E-05	0.003472	1.730749	1.955542283
730605	NM_020191.2	MRPS22	56945	MRPS22	7.025615	-3.199536	0.002495	0.02432	-1.699698	1.953839796
2320289	NM_005271.1	GLUD1	2746	GLUD1	8.141667	-2.913376	0.005504	0.039007	-2.407732	1.952416948
2600066	NM_001005849.1	SUMO2	6613	SUMO2	6.502463	-3.192911	0.002543	0.024514	-1.716564	1.95005742
5420053	NR_003110.2	CCT6P1	643253	CCT6P1	6.501972	-3.807458	0.000414	0.008851	-0.067836	1.948157143
1740220	NM_004038.3	AMY1A	276	AMY1A	5.734235	-3.621415	0.000728	0.011827	-0.583718	1.946926842
7150475	NM_017491.3	WDR1	9948	WDR1	8.562441	-3.456712	0.001188	0.015852	-1.02881	1.946055007

2900187	NM_001009551.1	CNIH	10175	CNIH	6.934849	-3.339636	0.001671	0.019557	-1.33803	1.940035446
650709	NM_025126.2	RNF34	80196	RNF34	6.549206	-4.021445	0.000213	0.006216	0.541125	1.940013261
830039	NM_001031711.1	ERGIC1	57222	ERGIC1	6.312664	-3.866702	0.000345	0.008061	0.099163	1.938487124
620240	NM_021962.2	ABR	29	ABR	7.015239	-2.997984	0.004374	0.034003	-2.20289	1.93726686
3370487	NM_004499.3	HNRNPAB	3182	HNRNPAB	6.30172	-4.473831	5.02E-05	0.003323	1.875182	1.93614705
1780402	NM_206926.1	SEPN1	57190	SEPN1	6.993683	-2.847984	0.006559	0.043407	-2.563338	1.935709513
4560092	NM_017737.3	FNBP1L	54874	FNBP1L	6.956805	-2.854726	0.006442	0.043045	-2.547406	1.933488585
3140167	NM_007145.1	ZNF146	7705	ZNF146	6.49127	-3.13716	0.002975	0.027024	-1.857644	1.933027077
7610338	NM_001793.3	CDH3	1001	CDH3	5.997042	-2.892458	0.005823	0.040214	-2.457771	1.932332756
2340092	NM_005648.2	TCEB1	6921	TCEB1	8.74057	-3.089404	0.003399	0.029175	-1.977244	1.93001001
1850047	NM_006589.2	FAM189B	10712	FAM189B	6.471756	-3.545596	0.000914	0.013644	-0.790028	1.928620607
6860373	NM_021932.4	RIC8A	60626	RIC8A	6.483633	-4.007152	0.000223	0.006423	0.499966	1.925031956
1110722	NM_018321.3	BRIX1	55299	BRIX1	6.029967	-3.197775	0.002508	0.024379	-1.704182	1.923172947
2710192	NM_007208.2	MRPL3	11222	MRPL3	7.991325	-3.01465	0.004179	0.033147	-2.162085	1.922772108
130608	NM_003626.2	PPFIA1	8500	PPFIA1	6.753809	-3.464041	0.001163	0.015624	-1.009251	1.921229884
770561	NM_080821.1	C20ORF108	116151	C20orf108	6.506843	-3.121085	0.003112	0.027836	-1.898033	1.919091561
2600035	NM_005381.2	NCL	4691	NCL	8.178577	-3.094595	0.00335	0.028981	-1.964301	1.917416835
10685	NM_058172.3	ANTXR2	118429	ANTXR2	5.972932	-3.939188	0.000276	0.007231	0.30518	1.916837389
3060035	NM_005826.3	HNRNPR	10236	HNRNPR	7.445274	-3.742346	0.000505	0.00969	-0.249894	1.916559246
1690114	NM_007279.2	U2AF2	11338	U2AF2	6.701353	-4.407434	6.23E-05	0.003573	1.675889	1.915932242
6110204	NM_013255.3	MKLN1	4289	MKLN1	6.399002	-4.161438	0.000137	0.005264	0.947668	1.915731031
3460754	NM_181050.1	AXIN1	8312	AXIN1	6.025839	-5.827418	5.26E-07	0.000446	6.106465	1.915060777
3850632	NM_138565.1	CTTN	2017	CTTN	6.303166	-4.059082	0.000189	0.006009	0.64982	1.912271047
6270706	NM_014837.3	SMG7	9887	SMG7	6.495739	-4.834468	1.53E-05	0.001854	2.975045	1.910480622
6510528	NM_017966.4	VPS37C	55048	VPS37C	8.210489	-2.925109	0.005333	0.038379	-2.379561	1.909120561
5960739	NM_002810.1	PSMD4	5710	PSMD4	7.945139	-2.910015	0.005554	0.039098	-2.415789	1.906804864
6940408	NM_015456.2	COBRA1	25920	COBRA1	6.684005	-3.838053	0.000377	0.008512	0.018249	1.905260939
6280482	NM_016101.3	NIP7	51388	NIP7	6.216969	-4.594863	3.38E-05	0.002738	2.24118	1.901612002
6220086	NM_001037494.1	DYNLL1	8655	DYNLL1	8.569031	-3.106608	0.00324	0.028581	-1.934292	1.900873591
5690333	NM_003400.3	XPO1	7514	XPO1	7.781944	-3.195575	0.002524	0.024435	-1.709783	1.900336227
1440343	NM_032842.2	TMEM209	84928	TMEM209	6.245283	-4.3558	7.36E-05	0.0039	1.521676	1.897272144
2360519	NM_003859.1	DPM1	8813	DPM1	6.381683	-3.718989	0.000543	0.009971	-0.314812	1.895945348
1820706	NM_178867.3	SFXN4	119559	SFXN4	6.654377	-2.987771	0.004498	0.0346	-2.227824	1.89554402
2600343	NM_014866.1	SEC16A	9919	SEC16A	6.969385	-3.226876	0.002309	0.023336	-1.629858	1.894312471
7400719	NM_016046.3	EXOSC1	51013	EXOSC1	7.170378	-3.02195	0.004096	0.032761	-2.144164	1.892929048
1240243	NM_015459.3	ATL3	25923	ATL3	6.424815	-2.780413	0.007843	0.048331	-2.721572	1.892046533
2710196	NM_018428.2	UTP6	55813	UTP6	6.38745	-4.106009	0.000163	0.005618	0.785974	1.889795234
6100382	NM_001015055.1	RTKN	6242	RTKN	6.396998	-3.806616	0.000415	0.008851	-0.070199	1.881408545
2970739	XR_038849.1	LOC100128353	100128353	LOC100128353	7.263022	-3.010985	0.004221	0.033333	-2.17107	1.879371446
2690215	NM_015607.2	C1ORF77	26097	C1orf77	6.385105	-5.180658	4.79E-06	0.001081	4.053421	1.877934904
5570196	NM_001005386.1	ACTR2	10097	ACTR2	7.343974	-2.997471	0.00438	0.034003	-2.204145	1.870450662
2100524	NM_025137.2	SPG11	80208	SPG11	6.324218	-4.602138	3.3E-05	0.002731	2.263289	1.869273106
6040112	NM_001384.4	DPH2	1802	DPH2	6.286567	-4.068334	0.000184	0.005981	0.67661	1.868777272
840528	NM_033452.2	TRIM47	91107	TRIM47	6.055448	-3.590961	0.000798	0.012556	-0.666872	1.865928768
2340180	NM_003564.1	TAGLN2	8407	TAGLN2	7.041061	-3.229244	0.002294	0.023261	-1.623792	1.865438135
5090020	NM_015327.1	SMG5	23381	SMG5	6.025791	-5.141432	5.47E-06	0.00113	3.930323	1.86483077
6020377	NM_001011551.1	C1GALT1C1	29071	C1GALT1C1	6.943274	-3.118517	0.003134	0.027948	-1.904471	1.864242791
5560180	NM_014933.2	SEC31A	22872	SEC31A	7.611561	-3.044725	0.003847	0.031426	-2.088071	1.864213698
1340491	NM_004510.2	SP110	3431	SP110	6.009727	-4.264278	9.88E-05	0.004548	1.250068	1.863273363
1740487	NM_004332.1	BPHL	670	BPHL	6.14864	-3.473465	0.001131	0.015468	-0.984065	1.863101354
7400673	NM_001039457.1	ATP6V0B	533	ATP6V0B	6.636102	-3.492375	0.00107	0.01506	-0.933411	1.863092902
1820470	NM_016167.3	NOL7	51406	NOL7	8.076201	-2.962982	0.004812	0.035908	-2.288107	1.858910631
1470259	NM_015219.2	EXOC7	23265	EXOC7	6.97879	-2.844866	0.006613	0.043509	-2.570698	1.858656889
1260193	NM_015470.1	RAB11FIP5	26056	RAB11FIP5	6.012329	-5.724906	7.49E-07	0.000494	5.778791	1.856361517
4760520	NM_014175.2	MRPL15	29088	MRPL15	6.421653	-2.855874	0.006422	0.042989	-2.544693	1.854769499
4560022	NM_015702.1	C2ORF25	27249	C2orf25	6.752096	-4.079973	0.000177	0.005909	0.710348	1.853686332
460129	NM_015654.3	NAT9	26151	NAT9	6.153433	-4.625429	3.06E-05	0.002713	2.334137	1.853518763
4150692	NM_017554.1	PARP14	54625	PARP14	5.903095	-4.255723	0.000102	0.004552	1.224796	1.852759559
2570692	NM_152713.2	STT3A	3703	STT3A	6.134169	-2.816922	0.007123	0.045597	-2.636404	1.852370201
3460201	NM_015999.2	ADIPOR1	51094	ADIPOR1	6.403986	-3.675752	0.000618	0.010654	-0.434432	1.849590921
4260019	NM_016645.2	NGRN	51335	NGRN	8.409463	-2.947929	0.005013	0.036805	-2.324549	1.846071885
5270575	NM_033392.3	MAPK8IP3	23162	MAPK8IP3	6.554987	-3.15332	0.002843	0.026232	-1.816911	1.843356778
7550411	NM_015043.3	TBC1D9B	23061	TBC1D9B	6.157048	-4.546268	3.96E-05	0.00288	2.093823	1.843201615
2360376	NM_024959.2	SLC24A6	80024	SLC24A6	6.34135	-3.725013	0.000533	0.00994	-0.29809	1.843126345
2900450	NM_015840.2	ADAR	103	ADAR	8.909948	-3.077631	0.003512	0.029711	-2.006549	1.841004975
3930070	NM_006842.2	SF3B2	10992	SF3B2	6.74184	-3.503226	0.001036	0.014804	-0.904274	1.838919769

1430187	NM_000100.2	CSTB	1476	CSTB	9.741053	-3.099343	0.003306	0.028849	-1.952449	1.837274387
4050370	NM_014168.1	METTL5	29081	METTL5	6.352121	-3.746273	0.000499	0.009647	-0.23896	1.837250412
7380634	NM_018270.3	C20ORF20	55257	C20orf20	6.622065	-4.555484	3.85E-05	0.00288	2.121728	1.835879605
6040114	NM_178000.1	PPP2R4	5524	PPP2R4	6.530026	-4.216129	0.000115	0.004766	1.108105	1.834522095
7100376	XR_018747.2	LOC345041	345041	LOC345041	7.385385	-2.826701	0.00694	0.045036	-2.613463	1.834096681
3890681	NM_017836.3	SLC41A3	54946	SLC41A3	6.545238	-3.56373	0.000866	0.013264	-0.7409	1.831963675
4730184	NM_018145.1	FAM82A2	55177	FAM82A2	6.724175	-3.697315	0.000579	0.010331	-0.374868	1.831807871
7200670	NM_016640.3	MRPS30	10884	MRPS30	6.212591	-2.904794	0.005633	0.039385	-2.428291	1.830775042
6900195	NM_018366.2	CNO	55330	CNO	6.299131	-4.148994	0.000143	0.005334	0.911286	1.8255061
4830056	NM_030978.1	ARPC5L	81873	ARPC5L	6.523771	-4.526565	4.23E-05	0.003014	2.034231	1.823210865
1510440	NM_002945.2	RPA1	6117	RPA1	6.846185	-3.63659	0.000696	0.011521	-0.542146	1.822766993
3520743	NM_012396.2	PHLDA3	23612	PHLDA3	6.301708	-3.117444	0.003143	0.027957	-1.907161	1.821946829
2690601	NM_001024959.1	ARPC4	10093	ARPC4	6.596773	-3.711055	0.000556	0.010074	-0.336818	1.820467895
6960440	NM_181762.1	UBE2A	7319	UBE2A	6.157361	-4.055254	0.000192	0.006048	0.638744	1.819395777
5390372	NM_001008215.1	C2ORF64	493753	C2orf64	6.224287	-5.394801	2.32E-06	0.000721	4.728798	1.819286217
450475	NM_001640.3	APEH	327	APEH	7.487187	-3.207212	0.002442	0.024089	-1.680125	1.819124791
5820463	NM_007166.2	PICALM	8301	PICALM	6.668875	-4.211806	0.000117	0.004766	1.095392	1.819084934
1050039	NM_007198.2	PROSC	11212	PROSC	6.359589	-2.793728	0.007573	0.047444	-2.690602	1.818874722
2260615	NM_004698.1	PRPF3	9129	PRPF3	6.26097	-3.98831	0.000237	0.006686	0.44581	1.818550401
1990630	NM_021158.3	TRIB3	57761	TRIB3	6.1589	-3.195191	0.002526	0.024435	-1.710762	1.818289468
3390592	NM_015530.3	GORASP2	26003	GORASP2	7.239149	-3.160737	0.002784	0.026003	-1.798172	1.817392881
60400	NM_002887.3	RARS	5917	RARS	8.364485	-2.913395	0.005504	0.039007	-2.407687	1.817144636
5810630	NM_153681.1	PIGP	51227	PIGP	6.646049	-3.415805	0.001339	0.017008	-1.137554	1.817081398
4810286	NM_019895.1	CLDND1	56650	CLDND1	6.600808	-3.440573	0.001246	0.016221	-1.0718	1.816853166
7560025	NM_148976.1	PSMA1	5682	PSMA1	8.507458	-3.349615	0.001624	0.019271	-1.311915	1.814811202
780762	NM_139062.1	CSNK1D	1453	CSNK1D	6.391258	-3.527227	0.000965	0.014132	-0.839652	1.814787379
7570358	NM_000271.1	NPC1	4864	NPC1	5.951301	-3.497182	0.001055	0.014948	-0.92051	1.810977572
3890092	NM_014781.3	RB1CC1	9821	RB1CC1	6.264393	-3.807955	0.000414	0.008851	-0.066441	1.810267858
5050082	NM_014281.3	PUF60	22827	PUF60	8.932507	-3.303076	0.001857	0.02065	-1.433308	1.809835221
5960358	NM_015946.4	PELO	53918	PELO	7.029463	-3.192115	0.002548	0.024523	-1.718589	1.80676377
5220187	NM_016454.2	TMEM85	51234	TMEM85	7.587145	-2.983925	0.004545	0.03486	-2.237197	1.806045096
10441	XR_018394.2	LOC440063	440063	LOC440063	6.134048	-2.775635	0.007942	0.048752	-2.732661	1.805863197
1410408	NM_032199.1	ARID5B	84159	ARID5B	6.173569	-3.6921	0.000589	0.010422	-0.38929	1.804315684
2570725	NM_001077447.1	PPCS	79717	PPCS	6.863594	-3.246286	0.002185	0.022656	-1.580058	1.803970339
4200162	NM_032830.1	CIRH1A	84916	CIRH1A	6.470297	-3.841767	0.000373	0.00844	0.028722	1.803107968
2450064	NM_006164.2	NFE2L2	4780	NFE2L2	6.070122	-4.965995	9.87E-06	0.001395	3.382501	1.80123947
5270768	NM_031268.4	PDPK1	5170	PDPK1	6.384048	-4.788732	1.78E-05	0.001953	2.834085	1.798764454
6060731	NM_012325.1	MAPRE1	22919	MAPRE1	7.241804	-3.197781	0.002508	0.024379	-1.704166	1.797755581
2120544	NM_018285.2	IMP3	55272	IMP3	7.763399	-3.030758	0.003998	0.032247	-2.122503	1.797284763
5700259	NM_014947.3	FOXJ3	22887	FOXJ3	6.24592	-4.462854	5.2E-05	0.003374	1.842161	1.795845884
2760491	NM_030803.5	ATG16L1	55054	ATG16L1	6.495979	-4.696611	2.42E-05	0.002336	2.551393	1.795760106
6510546	NM_020357.1	PCNP	57092	PCNP	8.007948	-3.045673	0.003837	0.031426	-2.085732	1.794552224
3290224	NM_182972.2	IRF2BP2	359948	IRF2BP2	5.984614	-4.813481	1.64E-05	0.001927	2.910316	1.790836298
6370164	NM_015344.1	LEPROTL1	23484	LEPROTL1	6.140405	-3.616083	0.00074	0.011962	-0.598306	1.789379654
2750551	NM_002455.2	MTX1	4580	MTX1	6.411917	-3.96342	0.000256	0.006921	0.374452	1.788267515
6660286	NM_017864.2	INTS8	55656	INTS8	5.861024	-5.170448	4.96E-06	0.001081	4.02136	1.785892
1090523	NM_001005861.1	RYK	6259	RYK	6.588715	-3.357525	0.001587	0.018955	-1.291184	1.784959311
4390195	NM_004965.6	HMGN1	3150	HMGN1	6.588904	-4.068941	0.000184	0.005981	0.678367	1.784950199
2810082	NM_016470.6	C20ORF111	51526	C20orf111	6.483841	-5.043701	7.6E-06	0.001172	3.624574	1.781902315
360475	NM_001005376.1	PLAUR	5329	PLAUR	5.878651	-3.164858	0.002752	0.025797	-1.787746	1.778313355
5960546	NM_015636.2	EIF2B4	8890	EIF2B4	7.065459	-3.792901	0.000433	0.009052	-0.108675	1.776884279
6520201	NM_032815.3	NFATC2IP	84901	NFATC2IP	6.701512	-3.778055	0.000453	0.009172	-0.150245	1.776203632
6110446	NM_002810.2	PSMD4	5710	PSMD4	6.239378	-4.127564	0.000153	0.005367	0.848742	1.77609607
2450446	NM_033020.2	TRIM33	51592	TRIM33	6.186762	-3.752006	0.000491	0.009553	-0.222984	1.77593663
2350703	NM_004177.3	STX3	6809	STX3	6.046204	-4.12618	0.000153	0.005367	0.844706	1.774409962
840148	NM_004068.3	AP2M1	1173	AP2M1	6.482186	-3.484087	0.001096	0.015269	-0.955632	1.773547386
610138	NM_002794.3	PSMB2	5690	PSMB2	7.699357	-2.844256	0.006624	0.043509	-2.572137	1.771904659
380402	NM_001105079.1	FBRS	64319	FBRS	6.222402	-5.478443	1.74E-06	0.00066	4.993954	1.771897779
460072	NM_006554.3	MTX2	10651	MTX2	7.298087	-2.880743	0.006009	0.040978	-2.485686	1.771215429
160669	NM_198398.1	ERGIC3	51614	ERGIC3	6.467573	-2.815252	0.007154	0.045756	-2.640317	1.770598696
7560097	NM_005765.2	ATP6AP2	10159	ATP6AP2	8.876774	-3.066235	0.003625	0.030361	-2.034844	1.769136841
3870543	NM_014676.2	PUM1	9698	PUM1	7.117681	-3.719226	0.000542	0.009971	-0.314154	1.767866779
7000431	NM_024569.3	MPZL1	9019	MPZL1	6.156415	-4.137112	0.000148	0.005334	0.876591	1.766826552
6330725	NM_005178.2	BCL3	602	BCL3	6.480767	-2.902659	0.005666	0.039447	-2.4334	1.765705034
2320402	NM_152499.1	CCDC24	149473	CCDC24	6.153795	-4.228846	0.000111	0.004716	1.145536	1.764528462
5290193	NM_153331.2	KCTD6	200845	KCTD6	6.022201	-4.448878	5.44E-05	0.00346	1.800156	1.76289238

6510437	NM_005968.3	HNRNPM	4670	HNRNPM	7.52517	-3.244912	0.002194	0.022672	-1.583588	1.762350897
6520497	NM_144600.1	C16ORF63	123811	C16orf63	6.480781	-3.869691	0.000342	0.008061	0.107623	1.76164344
6110474	NM_004494.1	HDGF	3068	HDGF	5.913588	-3.943261	0.000272	0.007174	0.316809	1.761478574
2850538	NM_012197.2	RABGAP1	23637	RABGAP1	6.248353	-3.306743	0.001838	0.020532	-1.423779	1.7605452
7050670	NM_014649.2	SAFB2	9667	SAFB2	6.236863	-4.782914	1.82E-05	0.001953	2.816185	1.759019177
6550341	NM_002027.2	FNTA	2339	FNTA	6.229153	-3.637499	0.000694	0.011521	-0.539653	1.758055523
4860093	XM_936251.1	LOC653888	653888	LOC653888	6.268919	-3.357806	0.001586	0.018955	-1.290446	1.757911923
5360653	NM_000098.1	CPT2	1376	CPT2	6.144906	-4.69757	2.41E-05	0.002336	2.554327	1.757715862
6960411	NM_002902.1	RCN2	5955	RCN2	6.31823	-4.222897	0.000113	0.004753	1.128022	1.757625004
3800398	NM_018438.4	FBXO6	26270	FBXO6	5.888205	-4.239288	0.000107	0.004633	1.176305	1.75670872
6020612	NM_002158.2	FOXN2	3344	FOXN2	6.169306	-3.81303	0.000407	0.008851	-0.052184	1.751225802
2350754	NM_017943.2	FBXO34	55030	FBXO34	6.113885	-3.266957	0.00206	0.022015	-1.52682	1.750087051
1260025	NM_016408.2	CDK5RAP1	51654	CDK5RAP1	6.011061	-5.723538	7.52E-07	0.000494	5.774424	1.748817614
2360471	NM_004893.2	H2AFY	9555	H2AFY	6.169726	-4.620191	3.11E-05	0.002713	2.318193	1.745791518
3190685	NM_017515.3	SLC35F2	54733	SLC35F2	6.073801	-3.250805	0.002157	0.02255	-1.568436	1.741381571
5130468	NM_032486.2	DCTN5	84516	DCTN5	6.633477	-3.230387	0.002286	0.023261	-1.620865	1.736358897
1090327	NM_032390.3	MKI67IP	84365	MKI67IP	6.13806	-3.684608	0.000602	0.010483	-0.409992	1.735859865
630333	NM_001008938.1	CKAP5	9793	CKAP5	6.526917	-3.155003	0.002829	0.026195	-1.812662	1.733643408
5890435	NM_000920.2	PC	5091	PC	5.994128	-2.801428	0.00742	0.046826	-2.672644	1.732900183
6200630	NM_018047.1	RBM22	55696	RBM22	8.302171	-3.135503	0.002989	0.027085	-1.861814	1.72984064
3840521	NM_032017.1	STK40	83931	STK40	7.203488	-2.790676	0.007634	0.04767	-2.69771	1.729401423
4880168	NM_004339.2	PTTG1IP	754	PTTG1IP	10.15178	-2.850858	0.006509	0.043271	-2.55655	1.729287474
450204	NM_024740.1	ALG9	79796	ALG9	5.995716	-3.036032	0.00394	0.031917	-2.109514	1.728795802
3290113	NM_001619.3	ADRBK1	156	ADRBK1	7.238287	-3.213924	0.002396	0.023915	-1.662988	1.72854145
4180142	NM_019600.1	KIAA1370	56204	KIAA1370	5.749958	-5.087016	6.57E-06	0.001172	3.759914	1.72458213
6020402	NM_004595.2	SMS	6611	SMS	6.626137	-3.602647	0.000771	0.012277	-0.635007	1.724134034
3360053	NM_006855.2	KDELR3	11015	KDELR3	6.36991	-2.869921	0.006186	0.041927	-2.511406	1.722619528
6060215	NM_016107.3	ZFR	51663	ZFR	6.601223	-3.658439	0.000652	0.011076	-0.482126	1.722193073
2940301	NM_024747.4	HPS6	79803	HPS6	6.702227	-3.424249	0.001307	0.016704	-1.115167	1.718557798
4920669	NM_005109.2	OXSR1	9943	OXSR1	6.076628	-4.548663	3.93E-05	0.00288	2.101074	1.71851076
2100241	NM_213589.1	RAPH1	65059	RAPH1	5.992895	-4.479039	4.94E-05	0.003304	1.890862	1.717540017
4780450	NM_005174.2	ATP5C1	509	ATP5C1	5.978697	-3.621889	0.000727	0.011827	-0.582421	1.716562073
770390	NM_006904.6	PRKDC	5591	PRKDC	6.034815	-3.789559	0.000438	0.009073	-0.118039	1.714195676
4780398	NM_013248.2	NXT1	29107	NXT1	6.710186	-2.907726	0.005589	0.039264	-2.421272	1.713330808
1340647	NM_032993.2	GAR1	54433	GAR1	7.008345	-2.941951	0.005095	0.037111	-2.338989	1.711674206
2490754	NM_022818.3	MAP1LC3B	81631	MAP1LC3B	6.351579	-2.979404	0.004602	0.035152	-2.248207	1.711381949
6960242	NM_019048.1	ASNSD1	54529	ASNSD1	6.08037	-5.954822	3.39E-07	0.00037	6.514448	1.711164495
4480280	NM_015695.1	BRPF3	27154	BRPF3	6.355744	-3.494301	0.001064	0.01503	-0.928243	1.710803072
6220367	NM_004135.2	IDH3G	3421	IDH3G	6.307976	-3.704459	0.000567	0.010205	-0.355092	1.709681759
520358	NM_003639.2	IKBKG	8517	IKBKG	6.083889	-3.683712	0.000604	0.010483	-0.412464	1.707651043
6960026	NM_006949.1	STXBP2	6813	STXBP2	6.658434	-2.926316	0.005315	0.038375	-2.376658	1.706927673
510487	NM_007111.3	TFDP1	7027	TFDP1	6.26215	-3.199858	0.002493	0.02432	-1.698876	1.706737549
4880563	NM_201274.2	MPRIP	23164	MPRIP	6.32604	-3.398437	0.001409	0.017428	-1.183498	1.703050418
6380131	NM_139353.1	TAF1C	9013	TAF1C	6.359955	-3.738688	0.000511	0.009718	-0.260075	1.70192988
2140762	NM_006761.3	YWHAE	7531	YWHAE	6.486965	-3.014196	0.004184	0.033154	-2.163197	1.701208025
7050136	NM_020673.2	RAB22A	57403	RAB22A	6.033305	-3.404257	0.001385	0.017303	-1.168116	1.701050209
4210129	NM_021814.3	ELOVL5	60481	ELOVL5	5.969385	-3.669213	0.000631	0.010842	-0.452461	1.700464775
6420446	NM_016308.1	CMPK1	51727	CMPK1	6.843218	-2.895641	0.005773	0.040016	-2.450172	1.698640428
940280	NM_030877.3	CTNNBL1	56259	CTNNBL1	5.859089	-2.867066	0.006233	0.042092	-2.518181	1.695811164
1450524	NM_015135.1	NUP205	23165	NUP205	6.557648	-3.119679	0.003124	0.027913	-1.901558	1.695742975
1470376	NM_020680.2	SCYL1	57410	SCYL1	6.151431	-3.69021	0.000592	0.010434	-0.394513	1.694330823
2370128	NM_032839.1	DIRC2	84925	DIRC2	6.221843	-3.426116	0.0013	0.016641	-1.110214	1.691580201
6550056	NM_207584.1	IFNAR2	3455	IFNAR2	5.947175	-5.044068	7.59E-06	0.001172	3.625721	1.691506893
6020474	NM_203472.1	SELS	55829	SELS	6.15186	-3.06436	0.003643	0.030384	-2.039494	1.690680204
5960278	NM_016312.2	WBP11	51729	WBP11	6.551961	-3.760388	0.000478	0.009432	-0.199605	1.686717101
6940242	NM_004634.2	BRPF1	7862	BRPF1	6.603725	-3.643941	0.000681	0.011417	-0.521974	1.686253723
5900735	NM_145868.1	ANXA11	311	ANXA11	5.773279	-3.795606	0.00043	0.009029	-0.101093	1.68622485
6660152	NM_021633.2	KLHL12	59349	KLHL12	6.113505	-4.0212	0.000213	0.006216	0.540419	1.685782684
3610709	NM_018440.3	PAG1	55824	PAG1	5.715539	-3.638051	0.000693	0.011521	-0.538139	1.685448904
5720327	NM_002788.2	PSMA3	5684	PSMA3	6.405978	-3.37987	0.001487	0.018131	-1.232463	1.684903246
2030070	NM_178167.2	ZNF598	90850	ZNF598	6.287499	-4.130723	0.000151	0.005339	0.857954	1.684472193
1030289	NM_005359.3	SMAD4	4089	SMAD4	6.125558	-4.554927	3.85E-05	0.00288	2.120042	1.683508298
2370167	XM_001717333.1	LOC100130154	100130154	LOC100130154	6.127645	-3.464012	0.001163	0.015624	-1.009329	1.682053396
6510427	NM_133368.1	RSPRY1	89970	RSPRY1	6.672625	-2.912773	0.005513	0.039007	-2.409179	1.681555292
6660600	NM_020312.1	COQ9	57017	COQ9	6.422471	-3.082085	0.003469	0.029509	-1.99547	1.68007616
2850441	NM_017798.2	YTHDF1	54915	YTHDF1	7.157332	-3.00778	0.004258	0.033475	-2.178922	1.67918578

5340414	NM_006893.2	LGTN	1939	LGTN	6.408088	-3.782322	0.000447	0.009103	-0.138304	1.677616489
7320743	NM_017730.2	QRICH1	54870	QRICH1	6.044183	-4.85896	1.41E-05	0.001737	3.05069	1.675109138
6270646	NM_002467.3	MYC	4609	MYC	6.077572	-2.789145	0.007665	0.047676	-2.701274	1.674140297
6040324	NM_001007067.1	SDCBP	6386	SDCBP	6.117818	-3.190579	0.002559	0.024599	-1.722496	1.673946528
5360221	NM_003372.4	VBP1	7411	VBP1	6.41811	-2.955621	0.00491	0.036323	-2.305941	1.673784451
10170	NM_005274.1	GNG5	2787	GNG5	6.149779	-4.013872	0.000218	0.006337	0.51931	1.673765399
5860037	NM_020765.1	UBR4	23352	UBR4	6.027363	-5.490101	1.67E-06	0.00066	5.030965	1.672956133
1240142	NM_017654.2	SAMD9	54809	SAMD9	5.725405	-2.979818	0.004597	0.035147	-2.247201	1.671269294
5890102	NM_021204.1	ENOPH1	58478	ENOPH1	6.220925	-4.925363	1.13E-05	0.001541	3.256311	1.669901009
7210327	NM_020792.3	AADACL1	57552	AADACL1	6.168274	-3.27238	0.002028	0.021885	-1.51282	1.669398196
1070754	NM_006367.2	CAP1	10487	CAP1	9.813594	-3.92618	0.000287	0.007303	0.268074	1.668411391
2600500	NM_004892.3	SEC22B	9554	SEC22B	6.324274	-3.381148	0.001482	0.018093	-1.229097	1.667822679
620458	NM_003074.2	SMARCC1	6599	SMARCC1	6.616649	-2.812266	0.007211	0.045932	-2.64731	1.667587852
5130458	NM_019030.2	DHX29	54505	DHX29	6.08247	-3.787444	0.00044	0.009089	-0.123965	1.667011266
6100441	NM_004697.3	PRPF4	9128	PRPF4	5.988205	-4.076958	0.000179	0.005917	0.701604	1.666197772
7100382	NM_025106.2	SPSB1	80176	SPSB1	5.844805	-5.693087	8.35E-07	0.000494	5.677212	1.665789708
3800487	NM_021035.1	ZNFX1	57169	ZNFX1	5.78418	-3.929181	0.000284	0.007284	0.27663	1.663692332
7380653	NM_019082.2	DDX56	54606	DDX56	6.66237	-2.853302	0.006466	0.043132	-2.550774	1.661268429
770326	NM_001135239.1	LDHA	3939	LDHA	10.60244	-2.855429	0.00643	0.043002	-2.545744	1.661253287
2850402	NM_005022.2	PFN1	5216	PFN1	9.566992	-3.431685	0.001279	0.01651	-1.095427	1.660519614
6290068	NM_015853.3	UBXN1	51035	UBXN1	7.383629	-2.817003	0.007121	0.045597	-2.636216	1.660461971
3140110	NM_015476.2	C18ORF10	25941	C18orf10	5.708038	-4.058606	0.00019	0.006009	0.648441	1.660209294
5810440	NM_031266.2	HNRNPAB	3182	HNRNPAB	9.06422	-2.874897	0.006104	0.041551	-2.499588	1.658877193
7560594	NM_022826.2	7-Mar	64844	MARCH7	7.057245	-3.472232	0.001135	0.015487	-0.987363	1.658335487
6380241	NM_018671.2	UNC45A	55898	UNC45A	6.288751	-3.823126	0.000395	0.00877	-0.023794	1.658022907
7160100	NM_015062.3	PPRC1	23082	PPRC1	6.042203	-4.046672	0.000197	0.006067	0.613929	1.657629822
2510750	NM_012308.1	FBXL11	22992	FBXL11	6.041294	-5.197331	4.53E-06	0.00108	4.105802	1.656063965
50114	NM_015190.3	DNAJC9	23234	DNAJC9	6.135671	-3.933797	0.00028	0.007239	0.289795	1.655946401
4920474	NM_022489.1	INF2	64423	INF2	5.94344	-2.945539	0.005046	0.036918	-2.330325	1.654448331
6130193	NM_024092.1	TMEM109	79073	TMEM109	6.466973	-3.218817	0.002363	0.023661	-1.650483	1.654008363
1090301	NM_001035513.1	SDHC	6391	SDHC	5.93001	-3.948742	0.000268	0.007117	0.332467	1.653655796
70674	NM_014742.2	TM9SF4	9777	TM9SF4	6.561954	-3.896451	0.000315	0.007726	0.183493	1.652621232
2450280	NM_016505.2	ZCCHC17	51538	ZCCHC17	6.09168	-5.854018	4.8E-07	0.000446	6.191581	1.652242979
4480368	NM_032936.2	TMEM60	85025	TMEM60	6.387807	-2.852982	0.006472	0.043132	-2.55153	1.651871321
1570433	NM_015635.2	GAPVD1	26130	GAPVD1	6.08542	-3.543787	0.000919	0.013664	-0.79492	1.649888058
3710605	NM_033375.3	MYO1C	4641	MYO1C	6.009056	-3.986192	0.000238	0.006687	0.43973	1.649573768
6060014	NM_001135865.1	LOC100132247	100132247	LOC100132247	6.207405	-3.407739	0.001371	0.017262	-1.158907	1.649002853
4390113	NM_025154.2	UNC84A	23353	UNC84A	5.853779	-3.936562	0.000278	0.007239	0.297684	1.648141344
3060022	NM_153812.1	PHF13	148479	PHF13	6.384489	-4.678899	2.56E-05	0.002415	2.497233	1.647089127
7050189	NM_014502.3	PRPF19	27339	PRPF19	6.072718	-4.394024	6.5E-05	0.003596	1.635771	1.646072487
7650142	NM_021103.3	TMSB10	9168	TMSB10	12.10852	-2.849739	0.006528	0.043271	-2.559195	1.644981944
5900600	NM_003418.1	CNBP	7555	CNBP	6.302002	-3.747313	0.000498	0.009641	-0.236061	1.640448178
4570500	NM_152931.1	CPNE1	8904	CPNE1	6.040592	-3.482255	0.001102	0.015269	-0.96054	1.640193812
5810070	NM_014814.1	PSMD6	9861	PSMD6	8.496776	-2.830957	0.006862	0.044682	-2.60346	1.640032601
150397	NM_001571.2	IRF3	3661	IRF3	6.238047	-4.400903	6.36E-05	0.003595	1.656343	1.639581506
7000703	NM_016310.2	POLR3K	51728	POLR3K	5.962984	-3.463226	0.001166	0.015633	-1.011427	1.638674252
4150279	NM_021184.3	C6ORF47	57827	C6orf47	6.048256	-3.996626	0.000231	0.006585	0.469697	1.638505447
5670241	NM_017454.1	STAU1	6780	STAU1	6.012832	-4.256199	0.000101	0.004552	1.226202	1.637406873
4260414	NM_004893.2	H2AFY	9555	H2AFY	9.812263	-3.713519	0.000552	0.010071	-0.329986	1.635718379
6130138	NM_006468.5	POLR3C	10623	POLR3C	6.75549	-2.903942	0.005646	0.039385	-2.43033	1.635535211
6770014	NM_003292.1	TPR	7175	TPR	6.3879	-3.210638	0.002418	0.023987	-1.671381	1.634756114
1470358	NM_024038.2	C19ORF43	79002	C19orf43	6.124054	-4.367029	7.1E-05	0.003814	1.555154	1.632831508
2600431	NM_017611.2	SLC43A3	29015	SLC43A3	5.76585	-3.453055	0.001201	0.01599	-1.038564	1.632082505
2490687	NM_004241.2	JMJD1C	221037	JMJD1C	6.152663	-4.003647	0.000225	0.006469	0.489884	1.631657276
20707	NM_014066.3	COMMD5	28991	COMMD5	6.040462	-4.223542	0.000113	0.004753	1.129919	1.631317552
1010168	NM_203433.1	PSMG1	8624	PSMG1	6.078441	-3.474504	0.001128	0.015448	-0.981287	1.630205734
4210431	NM_003135.1	SRP19	6728	SRP19	6.541609	-2.991068	0.004458	0.034463	-2.21978	1.628797063
6760576	NM_003169.2	SUPT5H	6829	SUPT5H	6.22681	-3.298065	0.001884	0.0208	-1.446318	1.628260362
6420296	NM_015950.3	MRPL2	51069	MRPL2	6.229119	-3.758235	0.000481	0.009452	-0.205613	1.627198792
6770161	NM_001038702.1	CDC42SE2	56990	CDC42SE2	5.99694	-4.052876	0.000193	0.006064	0.631867	1.625648521
6400474	NM_005439.1	MLF2	8079	MLF2	6.412348	-3.209546	0.002426	0.023995	-1.674169	1.625275673
7650091	NM_015308.1	FNBP4	23360	FNBP4	5.909402	-4.408328	6.21E-05	0.003573	1.678565	1.623495993
1580343	NM_014236.1	GNPAT	8443	GNPAT	6.267956	-3.799957	0.000424	0.008959	-0.088889	1.62243684
6040292	NM_015176.1	FBXO28	23219	FBXO28	5.998332	-3.908815	0.000303	0.007584	0.218633	1.62200067
4150689	XM_941466.1	METRNL	284207	METRNL	6.078803	-3.88089	0.00033	0.007928	0.139346	1.621989062
520040	NM_024595.1	AKIRIN1	79647	AKIRIN1	6.008118	-4.321976	8.21E-05	0.004161	1.421032	1.621119668

3850292	NM_144636.1	CHCHD4	131474	CHCHD4	6.216453	-3.7912	0.000435	0.009052	-0.113441	1.620051209
3520288	NM_001032279.1	RCE1	9986	RCE1	5.973514	-5.291463	3.29E-06	0.000866	4.402212	1.619743429
6100703	NM_002880.2	RAF1	5894	RAF1	6.023561	-4.787019	1.79E-05	0.001953	2.828814	1.619507821
5360630	NM_013242.2	C16ORF80	29105	C16orf80	6.237705	-3.705642	0.000565	0.010193	-0.351816	1.618705375
3440452	NM_207195.1	ADAM15	8751	ADAM15	9.80692	-2.842867	0.006649	0.043587	-2.575414	1.618592787
1780647	NM_052853.3	ADCK2	90956	ADCK2	6.14775	-3.184443	0.002604	0.024935	-1.738093	1.616119838
1770730	NM_003971.3	SPAG9	9043	SPAG9	6.21228	-3.005272	0.004288	0.033523	-2.185063	1.616054354
5560020	NM_005134.2	PPP4R1	9989	PPP4R1	6.450461	-2.913688	0.0055	0.039007	-2.406986	1.61472101
1780681	NM_144732.1	HNRPUL1	11100	HNRPUL1	6.537215	-3.619752	0.000732	0.011861	-0.588271	1.61432078
7210273	NM_181469.1	ITGB4BP	3692	ITGB4BP	8.887443	-3.663884	0.000641	0.010968	-0.467139	1.613838185
3290703	NM_003193.2	TBCE	6905	TBCE	5.996389	-3.927832	0.000286	0.00729	0.272784	1.613733147
5570538	NM_005088.2	SFRS17A	8227	SFRS17A	6.177908	-2.869868	0.006187	0.041927	-2.511531	1.612953734
70047	XR_041767.1	ZC3H11B	643136	ZC3H11B	6.77351	-3.220202	0.002353	0.023599	-1.646942	1.612767817
2850273	NM_018312.3	SAPS3	55291	SAPS3	6.233251	-3.942808	0.000273	0.007174	0.315515	1.610952361
6660554	NM_000989.2	RPL30	6156	RPL30	11.5079	-2.794139	0.007564	0.047444	-2.689644	1.610857008
6520605	NM_005871.2	SMNDC1	10285	SMNDC1	6.034521	-4.400898	6.36E-05	0.003595	1.65633	1.610579061
5720128	NM_015697.5	COQ2	27235	COQ2	6.283397	-3.092009	0.003374	0.02903	-1.97075	1.610351373
1410348	NM_004707.2	ATG12	9140	ATG12	6.574636	-2.863785	0.006288	0.042351	-2.52596	1.61024888
5810068	NM_153253.28	SIPA1	6494	SIPA1	6.371481	-2.792893	0.007589	0.04747	-2.692546	1.609553905
3180132	NM_175921.4	C5ORF51	285636	C5orf51	6.151006	-4.23617	0.000108	0.004633	1.167114	1.60831291
430259	NM_001012329.1	CTNNBIP1	56998	CTNNBIP1	5.98598	-3.551861	0.000897	0.013524	-0.773071	1.605829136
5720424	NM_003873.3	NRP1	8829	NRP1	5.901819	-2.825233	0.006967	0.045134	-2.61691	1.605586668
780603	NR_002305.1	PDIA3P	171423	PDIA3P	5.842254	-3.551137	0.000899	0.013527	-0.775031	1.605535726
5310605	NM_145012.3	CCNY	219771	CCNY	6.671211	-3.099003	0.003309	0.028849	-1.953298	1.605073514
2190273	NM_001025091.1	ABCF1	23	ABCF1	6.048312	-3.908178	0.000304	0.007584	0.216822	1.604341321
7040553	NM_006643.2	SDCCAG3	10807	SDCCAG3	5.973074	-3.683338	0.000604	0.010483	-0.413498	1.603845144
60079	NM_006145.1	DNAJB1	3337	DNAJB1	6.05535	-3.384679	0.001467	0.017989	-1.219794	1.603832208
2970368	NM_004724.2	ZW10	9183	ZW10	6.094241	-3.518201	0.000991	0.014412	-0.863984	1.601987427
20022	NM_003131.2	SRF	6722	SRF	6.270076	-3.813325	0.000407	0.008851	-0.051354	1.601108077
7000142	NM_014647.1	KIAA0430	9665	KIAA0430	5.987522	-4.557629	3.82E-05	0.00288	2.128227	1.599441344
1850021	NM_145305.1	SLC25A43	203427	SLC25A43	5.884599	-3.866904	0.000345	0.008061	0.099736	1.599131386
4860468	NM_001177.3	ARL1	400	ARL1	6.200037	-3.272143	0.00203	0.021885	-1.513431	1.599100214
5700088	XM_938887.1	KLF11	8462	KLF11	6.240826	-3.228644	0.002298	0.023261	-1.625329	1.598647114
2630064	NM_018477.1	ACTR10	55860	ACTR10	6.420067	-3.469072	0.001146	0.01553	-0.995809	1.598235582
4150520	NM_003821.4	RIPK2	8767	RIPK2	6.088077	-3.518037	0.000992	0.014412	-0.864424	1.598139781
7550563	NM_015638.2	TRPC4AP	26133	TRPC4AP	6.328792	-3.438657	0.001253	0.016231	-1.076898	1.59660929
4120403	NM_025234.1	WDR61	80349	WDR61	6.310359	-2.777376	0.007906	0.048589	-2.728623	1.59552445
3930349	NM_001042631.1	SDHAF1	644096	SDHAF1	6.273243	-2.981581	0.004575	0.035013	-2.242908	1.595323289
450097	NM_017657.3	AFTPH	54812	AFTPH	6.051274	-3.71252	0.000553	0.010074	-0.332755	1.59449348
360731	NM_001110781.1	LOC728661	728661	LOC728661	6.057878	-3.82714	0.00039	0.0087	-0.012497	1.593996477
4480026	NM_021970.2	MAP2K1IP1	8649	MAP2K1IP1	6.55991	-3.127776	0.003054	0.027498	-1.881238	1.593212118
1940064	NM_023923.2	PHACTR4	65979	PHACTR4	6.161088	-3.874361	0.000337	0.008061	0.120845	1.593175355
7160433	NM_001079514.1	UBN1	29855	UBN1	6.624397	-3.809174	0.000412	0.008851	-0.063015	1.592976055
4730148	NM_004986.2	KTN1	3895	KTN1	6.130868	-3.064572	0.003641	0.030384	-2.038969	1.592073883
5690291	XR_016251.2	LOC642590	642590	LOC642590	6.972953	-2.849946	0.006524	0.043271	-2.558705	1.590449742
1980500	NM_144723.1	ZMAT2	153527	ZMAT2	6.137537	-3.187601	0.002581	0.024775	-1.730067	1.589295852
830379	NM_176805.1	MRPS11	64963	MRPS11	6.76082	-2.78273	0.007795	0.048146	-2.71619	1.589194637
4200343	NM_001033028.1	CYFIP1	23191	CYFIP1	5.942795	-4.043666	0.000199	0.006067	0.605243	1.588851699
1820750	NM_139266.1	STAT1	6772	STAT1	5.705063	-3.112645	0.003186	0.028214	-1.919185	1.588797261
6620750	NM_020119.3	ZC3HAV1	56829	ZC3HAV1	6.092559	-4.286807	9.19E-05	0.004372	1.316713	1.588444251
4760692	NM_152379.2	C1ORF131	128061	C1orf131	6.163608	-3.201374	0.002482	0.024264	-1.695013	1.585866603
3710373	NM_001269.2	SNHG3-RCC1	751867	SNHG3-RCC1	6.27684	-2.91593	0.005467	0.039007	-2.401608	1.585696139
3420139	NM_021824.2	NIF3L1	60491	NIF3L1	7.197519	-3.067967	0.003607	0.030256	-2.030548	1.583943146
4850296	NM_005334.1	HCFC1	3054	HCFC1	6.755642	-3.371929	0.001522	0.018408	-1.253357	1.582806249
5700735	NM_031458.1	PARP9	83666	PARP9	6.31747	-2.928871	0.005279	0.038151	-2.370512	1.582797234
7560201	NM_022451.9	NOC3L	64318	NOC3L	5.877679	-4.144871	0.000145	0.005334	0.899243	1.582779608
540221	NM_006170.2	NOP2	4839	NOP2	6.288076	-3.310378	0.001819	0.020438	-1.414329	1.582497421
5390561	NM_014044.4	UNC50	25972	UNC50	8.280984	-2.78473	0.007754	0.047969	-2.711544	1.581739677
6330279	NM_213619.1	ATP6V1H	51606	ATP6V1H	6.166848	-2.909709	0.005559	0.039098	-2.416523	1.581124192
2340082	NM_001550.2	IFRD1	3475	IFRD1	5.726142	-4.321727	8.21E-05	0.004161	1.420293	1.580912112
150148	NM_018174.4	MAP1S	55201	MAP1S	5.928836	-4.387696	6.64E-05	0.003644	1.616856	1.580633803
4830520	NM_182507.1	KRT80	144501	KRT80	5.811249	-3.314496	0.001797	0.020375	-1.403613	1.580566127
4780519	NM_016478.3	ZC3HC1	51530	ZC3HC1	6.261257	-3.440026	0.001248	0.016221	-1.073257	1.580182808
5310358	NM_022173.1	TIA1	7072	TIA1	5.855578	-4.098785	0.000167	0.005671	0.76497	1.579135313
4860458	NM_023079.2	UBE2Z	65264	UBE2Z	6.06536	-4.103835	0.000165	0.005619	0.779651	1.578432188
1190221	NM_020385.2	REXO4	57109	REXO4	6.050544	-3.610935	0.000752	0.012051	-0.612377	1.577892433

6650121	NM_152609.1	C1ORF71	163882	C1orf71	6.12727	-3.127146	0.003059	0.027498	-1.88282	1.577042457
7400743	NM_020954.2	KIAA1618	57714	KIAA1618	5.755194	-2.868949	0.006202	0.041959	-2.513714	1.57663114
2470079	NM_003457.2	ZNF207	7756	ZNF207	7.762446	-2.907427	0.005593	0.039264	-2.421989	1.576555711
2260619	NM_080875.1	MIB2	142678	MIB2	6.081468	-3.736559	0.000514	0.009718	-0.265997	1.576159614
6660709	NM_014639.2	TTC37	9652	TTC37	5.847371	-3.970786	0.00025	0.006908	0.395548	1.576147022
6110487	NM_016223.3	PACSIN3	29763	PACSIN3	6.187311	-3.046917	0.003823	0.031426	-2.082659	1.576097237
7150678	NM_005370.4	RAB8A	4218	RAB8A	6.26395	-4.265528	9.84E-05	0.004548	1.253761	1.575657498
6860113	NM_015456.2	COBRA1	25920	COBRA1	6.05956	-3.969146	0.000251	0.006908	0.390849	1.575504097
1450156	NM_002370.2	MAGOH	4116	MAGOH	5.884688	-4.507268	4.5E-05	0.003123	1.975955	1.573933221
6420180	NM_080604.1	TJAP1	93643	TJAP1	6.206863	-3.202488	0.002475	0.024242	-1.692175	1.573918155
4850497	NM_006362.4	NXF1	10482	NXF1	6.207543	-3.587099	0.000807	0.012622	-0.677387	1.573245097
780598	NM_013254.2	TBK1	29110	TBK1	6.138262	-3.943831	0.000272	0.007174	0.318435	1.572962279
1940228	NM_152274.2	FAM58A	92002	FAM58A	5.81927	-4.630463	3.01E-05	0.002713	2.349468	1.571997696
4900484	NM_016052.2	RRP15	51018	RRP15	6.046826	-3.949858	0.000267	0.007117	0.335657	1.571644691
2100292	NM_002893.2	RBBP7	5931	RBBP7	6.155324	-2.918842	0.005424	0.038826	-2.394617	1.57066788
60324	NM_000276.3	OCRL	4952	OCRL	5.870585	-3.812293	0.000408	0.008851	-0.054253	1.569878093
940338	NM_032280.1	ZCCHC9	84240	ZCCHC9	6.033614	-4.285747	9.22E-05	0.004372	1.313574	1.56968227
3610647	NM_001077664.1	URG4	55665	URG4	6.071761	-3.603499	0.000769	0.012271	-0.632683	1.567914704
3130079	NM_014239.2	EIF2B2	8892	EIF2B2	5.935529	-3.384243	0.001469	0.017989	-1.220945	1.567629562
4230619	NM_012198.2	GCA	25801	GCA	5.925685	-3.624114	0.000723	0.011807	-0.576333	1.567130486
6980010	NM_025205.3	MED28	80306	MED28	6.290045	-3.378194	0.001495	0.018162	-1.236876	1.566190843
5270435	NM_015033.2	FNBP1	23048	FNBP1	5.884381	-3.407324	0.001373	0.017262	-1.160006	1.564959492
770411	NM_015288.4	PHF15	23338	PHF15	6.152653	-3.045326	0.00384	0.031426	-2.086588	1.564504631
870041	NM_012437.3	SNAPIN	23557	SNAPIN	6.029807	-3.249534	0.002165	0.022555	-1.571706	1.56334115
5900445	NM_175609.1	ARFGAP1	55738	ARFGAP1	6.108179	-3.59217	0.000795	0.012556	-0.663578	1.562664179
4670603	NM_133171.2	ELMO2	63916	ELMO2	6.188438	-3.442705	0.001238	0.016175	-1.066129	1.562089688
110270	NM_003906.3	MCM3AP	8888	MCM3AP	6.285615	-3.761869	0.000476	0.009432	-0.195471	1.560792259
830653	NM_001493.1	GDI1	2664	GDI1	6.224589	-3.094771	0.003349	0.028981	-1.96386	1.560254903
3060300	NM_021009.3	UBC	7316	UBC	12.75312	-3.477167	0.001119	0.015382	-0.974161	1.560193321
4280136	NM_175573.1	ADRM1	11047	ADRM1	5.910541	-4.446487	5.49E-05	0.00346	1.792975	1.559149163
7380470	NM_148906.1	OSBPL9	114883	OSBPL9	6.375518	-2.817138	0.007118	0.045597	-2.635898	1.558965512
5960722	NM_006545.4	TUSC4	10641	TUSC4	6.411578	-3.271224	0.002035	0.021885	-1.515805	1.558748202
4810288	NM_030628.1	INTS5	80789	INTS5	5.837244	-3.54425	0.000917	0.013664	-0.793669	1.558597851
7160348	NM_002748.2	MAPK6	5597	MAPK6	5.995234	-3.268745	0.002049	0.021934	-1.522206	1.558068911
4260392	NM_015922.1	NSDHL	50814	NSDHL	6.12107	-3.415209	0.001342	0.017009	-1.139132	1.557991471
5810632	NM_014306.2	C22ORF28	51493	C22orf28	6.283067	-3.571682	0.000845	0.013085	-0.719314	1.557507168
3890544	NM_024057.2	NUP37	79023	NUP37	6.336964	-2.830229	0.006876	0.044687	-2.605171	1.55724342
4880408	NM_006378.2	SEMA4D	10507	SEMA4D	5.949667	-3.360086	0.001575	0.018931	-1.284466	1.557208512
4780446	NM_181336.2	LEMD2	221496	LEMD2	5.914472	-4.118592	0.000157	0.005448	0.822598	1.557204614
6510133	NM_133635.4	POFUT2	23275	POFUT2	6.007628	-3.761	0.000477	0.009432	-0.197899	1.556195094
6860382	NM_007346.2	OGFR	11054	OGFR	5.853275	-4.030673	0.000207	0.006187	0.567732	1.55573096
6200195	NM_052844.3	WDR34	89891	WDR34	5.947716	-3.04255	0.00387	0.03155	-2.093441	1.555579815
3290044	NM_017735.3	TTC27	55622	TTC27	5.962294	-3.90288	0.000309	0.007622	0.201757	1.555418427
6520561	NM_020859.1	SHRM	57619	SHRM	6.395302	-2.958688	0.004869	0.036178	-2.298514	1.554217174
3180196	NM_015545.2	PTCD1	26024	PTCD1	6.34972	-3.558499	0.000879	0.013339	-0.755085	1.553626324
1440091	XM_937953.1	GON4L	54856	GON4L	5.940332	-5.328397	2.9E-06	0.000821	4.5188	1.553295647
4540328	NM_018150.2	RNF220	55182	RNF220	5.956347	-4.570325	3.66E-05	0.002865	2.166708	1.552943599
2470259	NM_032807.3	FBXO18	84893	FBXO18	6.50236	-2.76578	0.00815	0.049685	-2.755492	1.551215834
4210762	NM_022163.2	MRPL46	26589	MRPL46	5.956228	-3.292482	0.001915	0.021022	-1.4608	1.550603604
1990327	NM_020040.3	TUBB4Q	56604	TUBB4Q	5.830146	-3.995241	0.000232	0.006585	0.465717	1.55047911
840402	NM_015001.2	SPEN	23013	SPEN	6.04905	-4.09069	0.000172	0.005766	0.741453	1.550037828
4180204	NM_006312.2	NCOR2	9612	NCOR2	6.328736	-3.318337	0.001777	0.0203	-1.393613	1.549032176
5390092	NM_015014.1	RBM34	23029	RBM34	5.759742	-4.165534	0.000135	0.005264	0.959652	1.547585996
1090288	NM_000318.1	PXMP3	5828	PXMP3	5.846577	-4.087966	0.000173	0.00579	0.733543	1.54751887
3890255	NM_001068.2	TOP2B	7155	TOP2B	6.444983	-2.993135	0.004432	0.034304	-2.214735	1.547504448
1340092	NM_007367.2	RALY	22913	RALY	5.844489	-3.506677	0.001025	0.014709	-0.894998	1.547345834
3930730	NM_031452.2	FAM103A1	83640	FAM103A1	5.9929	-4.311685	8.48E-05	0.004191	1.390473	1.54641256
5090647	NM_002807.2	PSMD1	5707	PSMD1	6.412985	-2.824239	0.006986	0.045177	-2.619243	1.546269233
50170	NM_016038.2	SBDS	51119	SBDS	5.993217	-4.796493	1.74E-05	0.001953	2.857979	1.545809426
1450102	NM_001025204.1	U2AF1	7307	U2AF1	5.925506	-4.3846	6.71E-05	0.003655	1.607607	1.54562825
1500711	NM_014876.3	JOSD1	9929	JOSD1	5.914666	-3.77552	0.000457	0.009172	-0.157334	1.544748576
3140202	NM_001012643.2	MYPOP	339344	MYPOP	6.358354	-3.006914	0.004268	0.033475	-2.181044	1.544361762
7210373	NM_002816.3	PSMD12	5718	PSMD12	6.148385	-3.260804	0.002096	0.02225	-1.542687	1.543997726
3170239	NM_004383.1	CSK	1445	CSK	6.613131	-3.444135	0.001233	0.016137	-1.062323	1.543321917
3130576	NM_012110.2	CHIC2	26511	CHIC2	6.179286	-3.253107	0.002143	0.022494	-1.562513	1.542982577
6560181	NM_152912.3	MTIF3	219402	MTIF3	6.064113	-3.426343	0.001299	0.016641	-1.109611	1.542355424

4780743	NM_000126.1	ETFA	2108	ETFA	6.50896	-3.22853	0.002298	0.023261	-1.625623	1.541825975
3310546	NM_001950.3	E2F4	1874	E2F4	6.085621	-3.96283	0.000256	0.006921	0.372763	1.541806994
4920156	NM_002056.1	GFPT1	2673	GFPT1	6.03234	-3.100504	0.003295	0.028849	-1.949549	1.541369078
4640484	NM_004779.4	CNOT8	9337	CNOT8	6.317746	-3.257486	0.002116	0.022257	-1.551238	1.541327735
3870255	NM_016076.2	PPPDE1	51029	PPPDE1	6.299526	-3.037702	0.003922	0.031838	-2.105399	1.541220548
7100424	NM_032569.2	N-PAC	84656	N-PAC	5.81481	-4.119249	0.000157	0.005448	0.824513	1.541104157
6660195	NM_173659.2	RPUSD3	285367	RPUSD3	5.88506	-4.428887	5.81E-05	0.003472	1.74016	1.54106843
3120053	NM_015888.3	HOOK1	51361	HOOK1	6.075596	-2.775365	0.007947	0.048752	-2.733289	1.540623237
4120279	NM_006051.2	APBB3	10307	APBB3	6.033818	-2.835492	0.00678	0.044298	-2.592789	1.540580749
3420450	NM_015349.1	KIAA0240	23506	KIAA0240	5.937224	-4.132481	0.00015	0.005334	0.863081	1.536811392
160279	NM_145648.1	SLC15A4	121260	SLC15A4	6.022742	-3.755952	0.000485	0.009463	-0.211981	1.536659689
5690279	NM_006567.2	FARS2	10667	FARS2	6.08511	-3.481504	0.001105	0.015269	-0.96255	1.536176
6200670	NM_145693.1	LPIN1	23175	LPIN1	5.951816	-3.170574	0.002708	0.025513	-1.773275	1.53603954
7380367	NM_152833.2	C9ORF69	90120	C9orf69	6.207478	-4.212292	0.000117	0.004766	1.09682	1.535929581
4200575	NM_014232.1	VAMP2	6844	VAMP2	5.879687	-3.273675	0.002021	0.021885	-1.509475	1.534690129
4120491	NM_152879.2	DGKD	8527	DGKD	5.98755	-2.90392	0.005646	0.039385	-2.430383	1.534500834
2350215	NM_021807.2	EXOC4	60412	EXOC4	6.053153	-4.103111	0.000165	0.005619	0.777544	1.534472742
2600470	NM_018051.2	WDR60	55112	WDR60	5.835563	-3.204015	0.002464	0.024199	-1.688279	1.533490903
2640088	NM_184041.1	ALDOA	226	ALDOA	6.010831	-2.89593	0.005769	0.040016	-2.449482	1.532664838
5900594	NM_001779.1	CD58	965	CD58	5.834038	-3.429983	0.001285	0.016553	-1.099948	1.53154237
2710475	NM_007062.1	PWP1	11137	PWP1	6.142784	-3.487152	0.001086	0.015266	-0.947416	1.531441739
1240064	NM_012482.3	ZNF281	23528	ZNF281	6.050522	-2.885872	0.005927	0.040675	-2.473474	1.530645432
7150274	NM_001017371.3	SP3	6670	SP3	6.067512	-4.717409	2.26E-05	0.002267	2.615067	1.529749651
4060768	NM_001918.1	DBT	1629	DBT	6.482026	-2.765011	0.008166	0.049734	-2.757269	1.528743082
6350114	NM_006833.4	COPS6	10980	COPS6	6.07602	-2.799114	0.007466	0.047006	-2.678044	1.528086546
1450333	NM_016248.2	AKAP11	11215	AKAP11	5.985456	-3.251925	0.00215	0.022539	-1.565555	1.528058053
770692	NM_016390.2	C9ORF114	51490	C9orf114	5.972777	-3.569769	0.00085	0.013133	-0.724509	1.527552455
2190537	XM_936269.1	WDR74	54663	WDR74	6.270596	-2.780644	0.007838	0.048331	-2.721036	1.527162373
4150132	NM_017514.2	PLXNA3	55558	PLXNA3	5.663797	-3.798119	0.000426	0.008985	-0.094045	1.527151615
6270554	NM_201545.1	LGALS8	3964	LGALS8	6.177424	-2.917008	0.005451	0.038983	-2.399021	1.526593791
6560020	NM_016094.2	COMMD2	51122	COMMD2	5.965078	-3.112077	0.003191	0.028215	-1.920607	1.525727641
2470427	NM_005789.2	PSME3	10197	PSME3	5.826099	-4.215839	0.000115	0.004766	1.107252	1.525335289
6270288	NM_003092.3	SNRPB2	6629	SNRPB2	5.867272	-3.794003	0.000432	0.009049	-0.105586	1.523074602
2750367	NM_021127.1	PMAIP1	5366	PMAIP1	5.637514	-3.269381	0.002046	0.021924	-1.520563	1.522378763
1820035	NM_032850.3	ZFYVE19	84936	ZFYVE19	6.276761	-3.465758	0.001157	0.015624	-1.004664	1.522302401
7040397	NM_130809.2	PRRC1	133619	PRRC1	6.335218	-3.648546	0.000671	0.011285	-0.509327	1.521623608
4810615	NM_014655.1	SLC25A44	9673	SLC25A44	5.891144	-3.452557	0.001203	0.01599	-1.03989	1.521430532
3850520	NM_001039619.1	PRMT5	10419	PRMT5	6.304092	-2.784588	0.007757	0.047969	-2.711872	1.519443111
110678	Hs.368255	KIAA0368	23392	KIAA0368	6.052967	-4.214672	0.000116	0.004766	1.103821	1.518173076
7100711	NM_001743.3	CALM2	805	CALM2	10.83076	-3.136277	0.002982	0.027058	-1.859866	1.517237653
3360112	NM_013390.1	TMEM2	23670	TMEM2	6.040351	-2.782575	0.007798	0.048146	-2.716552	1.516572518
840292	NM_022768.4	RBM15	64783	RBM15	6.121292	-3.179991	0.002637	0.025124	-1.749397	1.515823619
2190441	XR_015946.2	LOC729200	729200	LOC729200	6.209229	-3.181709	0.002624	0.025033	-1.745036	1.514973419
6450681	NM_001933.3	DLST	1743	DLST	5.928202	-4.062534	0.000187	0.006009	0.659811	1.514548273
5310427	NM_152716.1	PATL1	219988	PATL1	5.966797	-4.275689	9.52E-05	0.004485	1.283807	1.51389797
1820189	NM_001545.1	ICT1	3396	ICT1	6.278167	-3.034641	0.003955	0.032006	-2.112941	1.513664301
3870452	XR_018556.2	LOC644879	644879	LOC644879	6.074435	-3.039435	0.003903	0.03172	-2.101124	1.513557654
150706	NM_006759.3	UGP2	7360	UGP2	5.845778	-4.13296	0.00015	0.005334	0.864478	1.513545502
3870646	NM_024065.3	PDCL3	79031	PDCL3	5.939107	-3.686084	0.000599	0.010483	-0.405914	1.512971415
3780270	NM_012257.3	HBP1	26959	HBP1	5.972467	-3.586461	0.000809	0.012622	-0.679126	1.510514631
670554	NM_031485.2	GRWD1	83743	GRWD1	6.062683	-4.043009	0.000199	0.006067	0.603347	1.508555261
620731	NM_006415.2	SPTLC1	10558	SPTLC1	6.881944	-2.765689	0.008152	0.049685	-2.755701	1.508319201
1580521	NM_015528.1	RNF167	26001	RNF167	6.16422	-3.364713	0.001554	0.018768	-1.272319	1.508222916
4490010	NM_014992.1	DAAM1	23002	DAAM1	6.172339	-2.771211	0.008035	0.049167	-2.742916	1.507794896
4490341	NM_004595.2	SMS	6611	SMS	6.240132	-3.205953	0.002451	0.024129	-1.683338	1.507561621
6380128	NM_007096.1	CLTA	1211	CLTA	5.983363	-2.823785	0.006994	0.045193	-2.620309	1.507557572
7050523	NM_006595.2	API5	8539	API5	6.255387	-3.702794	0.00057	0.010218	-0.359702	1.507424695
1450064	NM_005789.2	PSME3	10197	PSME3	5.950746	-3.696115	0.000582	0.010344	-0.378186	1.507049278
5820020	NM_006793.2	PRDX3	10935	PRDX3	6.050792	-3.272667	0.002026	0.021885	-1.512078	1.506103967
4290382	XM_371542.4	TMEM131	23505	TMEM131	6.562045	-3.001623	0.004331	0.033721	-2.193992	1.50594056
5910609	NM_000418.2	IL4R	3566	IL4R	5.918214	-3.685801	0.0006	0.010483	-0.406695	1.505344376
6860681	NM_016097.2	IER3IP1	51124	IER3IP1	5.925218	-2.974796	0.00466	0.035351	-2.259418	1.504820373
6660086	NR_003665.1	LOC441089	441089	LOC441089	6.430973	-3.117973	0.003139	0.027948	-1.905837	1.503836606
3890274	NM_006268.3	DPF2	5977	DPF2	6.388701	-3.518373	0.000991	0.014412	-0.863521	1.502900139
1740204	NM_017736.3	THUMPD1	55623	THUMPD1	6.162964	-2.929586	0.005269	0.038145	-2.36879	1.502733526
3060450	NM_005778.1	RBM5	10181	RBM5	6.506014	-3.01951	0.004123	0.032891	-2.150155	1.502595338

6180204	NM_145059.2	FUK	197258	FUK	6.073721	-2.850297	0.006518	0.043271	-2.557877	1.501807541
1940180	NM_002800.4	PSMB9	5698	PSMB9	5.619816	-3.078045	0.003508	0.02971	-2.005519	1.50144416
4920095	NM_014048.3	MKL2	57496	MKL2	5.713029	-4.201401	0.000121	0.004875	1.064816	1.501179182
1690379	NM_003766.2	BECN1	8678	BECN1	5.858689	-3.312467	0.001808	0.020398	-1.408893	1.500603845
3140156	NM_139078.1	MAPKAPK5	8550	MAPKAPK5	5.975005	-3.732528	0.000521	0.009787	-0.277208	1.500363966
620487	NM_078487.2	CDKN2B	1030	CDKN2B	5.963803	2.975036	0.004657	0.035351	-2.258834	0.66168348
2600204	NM_014016.2	SACM1L	22908	SACM1L	6.621547	3.177447	0.002656	0.025178	-1.755851	0.655821435
670010	XM_939387.1	LOC650298	650298	LOC650298	6.017997	3.160328	0.002787	0.026003	-1.799205	0.653910313
4290259	NM_024661.2	CCDC51	79714	CCDC51	5.91676	2.921665	0.005383	0.038659	-2.387838	0.648927576
2070279	NM_017837.2	PIGV	55650	PIGV	6.10436	3.883342	0.000328	0.007893	0.146296	0.648582384
1570168	NM_198859.1	PRICKLE2	166336	PRICKLE2	5.991023	4.427771	5.83E-05	0.003472	1.736816	0.648092539
6040064	NM_001006115.2	IP6K1	9807	IP6K1	6.538403	3.306896	0.001837	0.020532	-1.423382	0.647816975
5670605	NM_139354.2	MATK	4145	MATK	5.719301	4.427032	5.84E-05	0.003472	1.734598	0.647029006
4780113	NM_031468.2	CALN1	83698	CALN1	5.882935	4.599521	3.33E-05	0.002731	2.255333	0.645795343
6290296	NM_004146.4	NDUFB7	4713	NDUFB7	9.013916	3.04454	0.003849	0.031426	-2.088529	0.643398702
1780563	NM_014494.1	TNRC6A	27327	TNRC6A	5.738198	3.872558	0.000339	0.008061	0.11574	0.640138324
5550068	NM_007152.1	ZNF195	7748	ZNF195	6.004329	3.361485	0.001569	0.018884	-1.280792	0.638491903
4040553	XM_936248.1	LOC642109	642109	LOC642109	5.759013	3.902956	0.000309	0.007622	0.201975	0.63398182
6200561	NM_024296.3	CCDC28B	79140	CCDC28B	5.878837	4.31336	8.44E-05	0.004191	1.395445	0.631521883
4490333	NM_024581.3	FAM184A	79632	FAM184A	5.950098	3.244338	0.002197	0.022672	-1.585064	0.629183758
3370762	NM_001013685.1	LOC401357	401357	LOC401357	6.079699	3.212623	0.002405	0.023955	-1.666312	0.62798782
540280	XM_927887.3	LOC644790	644790	LOC644790	9.197963	3.068836	0.003599	0.030256	-2.028393	0.626188299
7150711	NM_013361.2	ZNF223	7766	ZNF223	6.205888	3.450499	0.00121	0.016031	-1.045375	0.625600713
5810367	NM_018177.2	N4BP2	55728	N4BP2	6.564343	2.956287	0.004901	0.036309	-2.304329	0.622771369
3800431	NM_018254.2	RCOR3	55758	RCOR3	7.111777	2.789474	0.007658	0.047676	-2.700507	0.620183313
6100411	NM_017946.2	FKBP14	55033	FKBP14	5.955557	3.757998	0.000482	0.009452	-0.206275	0.619852326
6940202	XM_114618.6	R3HCC1	203069	R3HCC1	6.477787	3.412201	0.001354	0.017102	-1.147097	0.615890792
1190121	XM_001720643.1	LOC389765	389765	LOC389765	6.241169	3.33788	0.00168	0.019602	-1.342621	0.615156367
3460184	NM_014047.1	C19ORF53	28974	C19orf53	8.489116	2.820023	0.007064	0.045484	-2.629134	0.6146611
4180431	NM_174925.1	LOC205251	205251	LOC205251	6.727298	3.326769	0.001735	0.020057	-1.371634	0.611554446
6560148	NM_003198.1	TCEB3	6924	TCEB3	6.175067	4.068078	0.000184	0.005981	0.675869	0.609061793
6660487	NM_032679.1	ZNF577	84765	ZNF577	6.36299	3.857253	0.000355	0.008243	0.072444	0.608125264
5690017	NM_001033515.1	LOC100132288	100132288	LOC100132288	5.850763	3.867137	0.000345	0.008061	0.100396	0.607825586
5260360	XM_001726504.1	LOC100131801	100131801	LOC100131801	9.994164	3.493653	0.001066	0.015031	-0.929981	0.607803465
1430753	NM_006202.1	PDE4A	5141	PDE4A	6.056447	3.025218	0.004059	0.032525	-2.136133	0.606522318
2810632	NM_173176.1	PTK2B	2185	PTK2B	5.883947	4.13749	0.000148	0.005334	0.877693	0.604577317
3610114	NM_014186.1	COMMD9	29099	COMMD9	6.92137	3.209281	0.002428	0.023995	-1.674847	0.603404297
1340465	NM_033455.1	KCNK7	10089	KCNK7	5.724277	4.485935	4.83E-05	0.003288	1.911631	0.600988107
4260441	NM_003278.1	CLEC3B	7123	CLEC3B	5.634087	4.872688	1.35E-05	0.001686	3.093136	0.599302378
4260520	NM_006838.2	METAP2	10988	METAP2	9.017028	3.086408	0.003427	0.029269	-1.984708	0.599102113
7560575	NM_020422.3	TMEM159	57146	TMEM159	6.033389	2.876703	0.006074	0.041387	-2.495295	0.596066355
4880465	NM_014942.2	ANKRD6	22881	ANKRD6	6.001529	3.985899	0.000238	0.006687	0.43889	0.594804926
2320367	NM_015965.3	NDUFA13	51079	NDUFA13	8.639295	3.063988	0.003647	0.030384	-2.040417	0.593219177
5670315	NM_016199.1	LSM7	51690	LSM7	8.352916	2.845492	0.006602	0.043509	-2.569222	0.583190077
2640358	XM_001126276.1	LOC730288	730288	LOC730288	6.708452	3.48634	0.001089	0.015269	-0.949593	0.582407539
7380619	NM_007317.1	LOC728037	728037	LOC728037	5.95839	4.336005	7.84E-05	0.004072	1.462739	0.580644427
3870386	NM_005279.2	GPR1	2825	GPR1	6.494277	3.314029	0.0018	0.020375	-1.404829	0.578340705
1580576	NM_004867.3	ITM2A	9452	ITM2A	5.925951	4.261886	9.95E-05	0.004548	1.242998	0.577773907
4390768	NM_018907.2	PCDHA1	56147	PCDHA1	5.823711	5.263018	3.63E-06	0.000922	4.312528	0.570476781
2600072	NM_032515.3	BOK	666	BOK	6.686228	2.788	0.007688	0.047696	-2.703938	0.567667765
4900053	NM_001002251.1	ARL6IP4	51329	ARL6IP4	7.975456	3.010348	0.004228	0.033333	-2.172631	0.564281277
6060270	XM_001720255.1	LOC100129445	100129445	LOC100129445	5.923799	3.892909	0.000318	0.00776	0.173437	0.556824607
1170673	NM_172014.1	TNFSF14	8740	TNFSF14	6.599603	3.514407	0.001002	0.014485	-0.874199	0.556261839
2600576	XM_945812.1	PRNPIP	79033	PRNPIP	6.631766	4.317651	8.32E-05	0.004178	1.408184	0.555205094
5810735	NR_003543.1	LOC646996	646996	LOC646996	7.435054	3.016439	0.004158	0.033119	-2.157694	0.553828237
110075	NM_015049.1	TRAK2	66008	TRAK2	6.51313	3.270508	0.002039	0.021885	-1.517654	0.550388411
5490594	NM_001017391.1	SULT1A4	445329	SULT1A4	6.430162	3.600198	0.000776	0.012341	-0.641689	0.549321633
1010195	NM_001079863.1	DBI	1622	DBI	9.828865	3.684367	0.000603	0.010483	-0.410655	0.548013696
3780494	XR_038705.1	LOC100131718	100131718	LOC100131718	6.783576	3.471814	0.001137	0.015487	-0.988479	0.541813321
5690400	NR_003082.1	GSTTP2	653399	GSTTP2	5.992567	5.389081	2.36E-06	0.000721	4.710691	0.533697212
5290482	NM_031943.1	IFP38	83880	IFP38	6.929631	4.05178	0.000194	0.006064	0.628697	0.527918645
7330068	NM_031287.2	SF3B5	83443	SF3B5	8.240488	2.974611	0.004662	0.035351	-2.259867	0.526563498
70441	NM_024407.3	NDUFS7	374291	NDUFS7	8.561558	2.990623	0.004463	0.03447	-2.220867	0.526495483
2480338	NM_020548.4	DBI	1622	DBI	6.653405	3.128357	0.003049	0.027498	-1.879779	0.526220695
1050612	NM_177530.1	SULT1A1	6817	SULT1A1	8.308916	3.322223	0.001758	0.020226	-1.383486	0.517858033
2570433	NM_145341.2	PDCD4	27250	PDCD4	7.669421	3.010482	0.004227	0.033333	-2.172304	0.511515084

5870048	XM_934113.1	LOC653489	653489	LOC653489	7.725707	3.693146	0.000587	0.010414	-0.386398	0.504032927
1570370	NM_032593.2	HINT2	84681	HINT2	7.587493	3.48215	0.001102	0.015269	-0.960821	0.502754162
2810707	NM_005038.2	PPID	5481	PPID	6.080295	4.426631	5.85E-05	0.003472	1.733397	0.49762852
3780082	NM_144626.1	TMEM125	128218	TMEM125	8.71655	2.77366	0.007983	0.048931	-2.73724	0.49627668
5340059	XM_938862.1	LRRC58	116064	LRRC58	6.338996	3.971878	0.000249	0.006908	0.398678	0.493889443
1450377	NM_001487.1	BLOC1S1	2647	BLOC1S1	8.077346	3.087629	0.003416	0.029254	-1.981667	0.489119359
130561	NM_001512.2	GSTA4	2941	GSTA4	6.259946	3.09675	0.00333	0.02891	-1.958923	0.484079454
6510204	NM_006416.2	SLC35A1	10559	SLC35A1	7.400253	3.505822	0.001028	0.014718	-0.897298	0.476013075
1230349	XR_040489.1	LOC729652	729652	LOC729652	5.903735	5.112841	6.02E-06	0.001163	3.840736	0.474677349
7560041	NM_033027.2	AXUD1	64651	AXUD1	7.364329	3.161621	0.002777	0.025972	-1.795937	0.473017897
520059	NM_014398.2	LAMP3	27074	LAMP3	6.198913	3.0591	0.003697	0.030598	-2.052528	0.460628318
3520408	NM_001080113.1	PP8961	650662	PP8961	5.956713	4.729113	2.17E-05	0.00221	2.650938	0.45146123
1010364	NM_033257.2	DGCR6	8214	DGCR6	8.801914	3.44682	0.001223	0.016068	-1.055175	0.44908887
1010487	NM_006763.2	BTG2	7832	BTG2	6.563978	3.2706	0.002039	0.021885	-1.517417	0.436360995
4880626	NM_018326.2	GIMAP4	55303	GIMAP4	6.44935	2.873669	0.006124	0.04165	-2.502507	0.435694074
2850142	XR_038694.1	LOC100129650	100129650	LOC100129650	8.655955	3.650845	0.000667	0.011247	-0.503009	0.430312416
1110343	XM_001725987.1	LOC100132037	100132037	LOC100132037	9.278314	3.474735	0.001127	0.015448	-0.980667	0.426688685
6660575	NM_017548.3	LOC729692	729692	LOC729692	6.227915	4.789973	1.78E-05	0.001953	2.837906	0.423650796
1570523	NM_032204.3	ASCC2	84164	ASCC2	7.344214	3.829507	0.000387	0.008662	-0.005831	0.421592912
7200274	NM_005675.2	DGCR6	8214	DGCR6	8.637259	3.637146	0.000695	0.011521	-0.54062	0.420832393
1740458	NM_152793.1	C7ORF41	222166	C7orf41	6.644893	4.029006	0.000208	0.006187	0.562925	0.419359833
5270112	NM_002130.4	HMGCS1	3157	HMGCS1	7.6387	2.790369	0.00764	0.04767	-2.698425	0.417562846
1230546	NM_015012.1	TMEM41B	440026	TMEM41B	8.5453	4.177738	0.00013	0.005147	0.995394	0.415334475
2260132	NM_016063.1	HDDC2	51020	HDDC2	8.787539	4.603126	3.29E-05	0.002731	2.26629	0.408526753
2650598	NM_207352.2	CYP4V2	285440	CYP4V2	7.038937	4.05902	0.00019	0.006009	0.64964	0.387978121
6250292	XM_001722130.1	LOC646753	646753	LOC646753	6.988269	5.071887	6.92E-06	0.001172	3.712611	0.368896918
1240446	NM_001752.2	CAT	847	CAT	6.674429	5.53705	1.43E-06	0.000657	5.18014	0.364723651
3930392	NM_001145.2	ANG	283	ANG	7.700806	3.04728	0.00382	0.031426	-2.081762	0.35150884
5570201	NM_005426.2	TP53BP2	7159	TP53BP2	6.150816	6.317209	9.7E-08	0.000131	7.677598	0.349606299
1990468	NM_016824.3	ADD3	120	ADD3	7.684824	2.83801	0.006735	0.04404	-2.586861	0.349306636
6420008	NM_000313.1	PROS1	5627	PROS1	6.737335	2.965647	0.004778	0.035813	-2.281642	0.348166299
270326	XR_041791.1	LOC730176	730176	LOC730176	6.438136	4.890097	1.27E-05	0.001659	3.147013	0.345853108
4480148	NM_001113411.1	FGGY	55277	FGGY	6.735154	4.62126	3.1E-05	0.002713	2.321448	0.344910119
6270725	XM_936750.1	SULT1A1	6817	SULT1A1	7.02176	5.172763	4.92E-06	0.001081	4.028628	0.341540076
6020561	XM_930677.1	LOC642299	642299	LOC642299	7.101093	4.2056	0.000119	0.004836	1.077151	0.33662294
6550754	NM_016337.2	EVL	51466	EVL	8.124092	3.626114	0.000718	0.011762	-0.570855	0.332848261
7570768	XM_001715065.1	LOC100129543	100129543	LOC100129543	6.387923	5.552043	1.35E-06	0.000657	5.227818	0.32042684
7610553	NR_024609.1	FLJ44054	643365	FLJ44054	6.490596	5.539657	1.41E-06	0.000657	5.18843	0.318542659
6450010	XM_001723164.1	LOC100129952	100129952	LOC100129952	6.500048	6.443002	6.28E-08	0.00012	8.081665	0.293499946
2600767	XM_001724645.1	LOC100133568	100133568	LOC100133568	6.369251	4.697597	2.41E-05	0.002336	2.554409	0.291881535
7160475	XM_943699.1	C6ORF160	387066	C6orf160	8.88007	3.326826	0.001734	0.020057	-1.371486	0.29035733
1430280	NM_004364.2	CEBPA	1050	CEBPA	7.16763	3.587306	0.000807	0.012622	-0.676823	0.287250032
3120180	NM_001005505.1	CACNA2D2	9254	CACNA2D2	6.298039	5.345674	2.74E-06	0.000804	4.573392	0.284345445
940639	NM_152321.1	ERP27	121506	ERP27	6.951856	2.912628	0.005515	0.039007	-2.409527	0.276676678
2680056	NM_001025195.1	CES1	1066	CES1	6.436327	4.253361	0.000102	0.004552	1.217823	0.266817873
6270114	NM_172313.1	CSF3R	1441	CSF3R	6.868959	3.177725	0.002654	0.025178	-1.755148	0.265811397
2650164	NM_018291.2	FLJ10986	55277	FLJ10986	7.29322	4.026195	0.00021	0.006191	0.554818	0.262273476
5870161	XM_001717180.1	LOC100130701	100130701	LOC100130701	6.453448	6.90775	1.26E-08	5.41E-05	9.572089	0.262195641
5490019	NM_002084.2	GPX3	2878	GPX3	7.251011	3.726396	0.00053	0.009922	-0.294249	0.258548085
3870703	NM_016434.2	RTEL1	51750	RTEL1	6.754893	6.529178	4.66E-08	0.000119	8.35841	0.24640022
6510373	NM_001037330.1	TRIM16L	147166	TRIM16L	6.409094	5.040817	7.68E-06	0.001172	3.615573	0.241057241
7550445	NM_198477.1	CXCL17	284340	CXCL17	7.156639	4.048255	0.000196	0.006067	0.618504	0.215369873

Supplementary Table 3: LIMMA analysis revealed 815 differentially expressed non-redundant genes (FDR 5%, fold change > 1.5 or < 0.66) between solid and leipidic architecture specimens. The gene list is ordered according to a decreasing fold change.
