# Supplementary material for: Molecular signatures in IASLC/ATS/ERS classified growth patterns of lung adenocarcinoma
Source: PLoS One. 2018 Oct 23;13(10):e0206132. doi: 10.1371/journal.pone.0206132 (PMC6198952; doi:10.1371/journal.pone.0206132)
Supplement: S4 Table — shows the comparison of the 50 highest upregulated genes in solid or lepidic growth pattern with published gene signatures using GeneSigDB. Table view was restricted to studies with a minimum of 20% overlap (10 genes). (PDF) [file pone.0206132.s008.pdf]

Pattern	Gene Signature Link	Signature Name	Signature Size	Gene List Size	Overlap	Background	P-value
solid	15897907-SuppTable2	Breast_Farmer05_3198genes_basal_apocrine_luminal	3160	50	24	20523	0
solid	20174566-TableS1	Breast_Walker10_3588genes	3168	50	18	20523	0
solid	16872506-SuppTable1	Leukemia_Yukinawa06_2000genes	1549	50	18	20523	0
solid	20220088-SuppTable1	Immune_Allen10_2900genes	1926	50	16	20523	0
solid	18387200-Genes	Breast_Mutarelli08_1488genes	1212	50	15	20523	0
solid	20081105-ST-2	Breast_Nagaraja10_3054genes	2415	50	15	20523	0
solid	11823860-SuppTable3	Breast_van'tVeer02_2460genes_Ergenes	1995	50	13	20523	0
solid	19192944-TableS4	Ovarian_Crijns09_2394Genes_17PathwayPredictor	1586	50	13	20523	0
solid	18757322-TableS1	Breast_Creighton08_2154genes	1803	50	13	20523	0
solid	18631401-TableS3	Breast_Yau08_1518genes_OxidativeStress	1173	50	12	20523	0
solid	19843711-TableS1	Kidney_Sallustio10_2134genes_DiscriminatedARPCsFromRPTEC/MSC	1739	50	12	20523	0
solid	19843711-TableS2	Kidney_Sallustio10_2134genes_CompleteListAnalysis	1725	50	12	20523	0
solid	17724462-TableS1	StemCell_Nuytten08_1622genes	1449	50	11	20523	0
solid	17297478-SuppTable5	Intestine_Vecchi07_1024genes	818	50	10	20523	0
solid	18689800-TableS7	EmbryonicStemCell_Thomas08_1088genes	1026	50	10	20523	0
solid	12917485-Table8	Breast_Sotiriou03_706genes	521	50	10	20523	0
lepidic	15897907-SuppTable2	Breast_Farmer05_3198genes_basal_apocrine_luminal	3160	50	20	20523	0
lepidic	19218430-TableS3	StemCell_Majeti09_3024genes	2949	50	16	20523	0
lepidic	18757322-TableS1	Breast_Creighton08_2154genes	1803	50	12	20523	0

Supplementary Table 4: Comparison of the 50 highest upregulated genes in solid or lepidic growth pattern with published gene signatures using GeneSigDB. Table view was restricted to studies with a minimum of 20% overlap (10 genes).
