# Supplementary material for: Molecular signatures in IASLC/ATS/ERS classified growth patterns of lung adenocarcinoma
Source: PLoS One. 2018 Oct 23;13(10):e0206132. doi: 10.1371/journal.pone.0206132 (PMC6198952; doi:10.1371/journal.pone.0206132)
Supplement: S5 Table — Known lung adenocarcinoma oncogenes and tumor suppressor genes were shown with MEAN and SD expression values in all growth patterns. T-test between different growth patterns were mostly not significant (p-value > 0.05, not adjusted). Only one group comparison indicated higher expression of MET and MAP2K1 (MEK1) in solid compared to lepidic growth pattern (p-value 0.04). (PDF) [file pone.0206132.s009.pdf]

Probe_ID	Symbol	acinar		micropapillary		papillary		lepidic		solid		lepidic vs solid
		Mean	SD	Mean	SD	Mean	SD	Mean	SD	Mean	SD	P.Value
6330307	EGFR	100,9	14,9	106,6	16,7	104,9	21,5	93,0	8,2	108,4	31,8	0,65
1070673	ERBB2	86,3	5,8	86,0	6,1	80,1	7,1	81,7	4,8	86,0	3,9	0,87
4200504	MET	170,7	63,8	858,5	982,2	244,7	124,3	**108,7**	17,2	**490,8**	407,5	**0,04**
7210202	BRAF	84,1	5,8	89,6	11,3	79,2	4,3	75,8	5,1	98,2	38,0	0,47
4540100	KRAS	82,5	3,1	81,1	4,3	81,4	3,8	79,5	5,7	82,4	3,4	0,91
6020280	NRAS	95,9	17,9	107,6	32,3	90,1	12,0	80,5	3,4	140,7	84,2	0,20
7210424	AKT1	91,1	3,5	94,2	6,7	87,1	6,5	86,3	5,5	95,3	9,5	0,76
1050600	MAP2K1	190,9	82,3	314,6	217,2	239,1	161,2	**93,6**	10,8	**327,7**	224,6	**0,04**
1240520	PIK3CA	82,2	4,5	79,3	5,0	74,7	5,0	77,7	4,8	80,4	2,2	0,91
6250431	ALK	95,5	9,0	98,1	6,4	98,6	12,0	86,9	5,6	100,6	13,9	0,66
4180500	TP53	110,5	13,8	112,3	9,7	119,9	29,9	100,0	5,9	118,1	26,7	0,62
510452	FHIT	99,6	12,7	102,1	13,3	97,0	10,1	96,1	6,0	103,5	10,9	0,82
6350121	RASSF1	96,5	9,2	97,8	6,1	89,4	7,2	94,1	8,6	96,2	8,7	0,94
6620014	CDKN2A	102,9	28,0	107,8	20,8	107,0	20,3	91,8	8,8	105,2	34,9	0,69
5720441	RARB	82,4	3,2	83,1	8,8	78,7	4,4	77,4	4,4	88,9	11,0	0,67

Supplementary Table 5: Known lung adenocarcinoma oncogenes and tumor suppressor genes were shown with MEAN and SD expression values in all growth patterns. T-test between different growth patterns were mostly not significant (p-value > 0.05, not adjusted). Only one group comparison indicated higher expression of MET and MAP2K1 (MEK1) in solid compared to lepidic growth pattern (p-value 0.04).

acinar vs lepid	acinar vs micr	acinar vs papil	acinar vs solid	lepidic vs micr	lepidic vs papi	micropapillary	micropapillary	papillary vs solid
0,80	0,86	0,89	0,83	0,67	0,71	0,96	0,97	0,94
0,87	1,00	0,83	0,99	0,86	0,96	0,84	0,99	0,84
0,23	0,10	0,38	0,09	0,07	0,09	0,14	0,40	0,20
0,75	0,84	0,87	0,66	0,61	0,88	0,72	0,81	0,56
0,91	0,97	0,98	1,00	0,94	0,93	0,99	0,98	0,98
0,60	0,73	0,87	0,35	0,42	0,73	0,63	0,52	0,30
0,87	0,91	0,91	0,89	0,78	0,96	0,82	0,98	0,80
0,09	0,29	0,60	0,24	0,05	0,09	0,57	0,94	0,51
0,86	0,93	0,79	0,95	0,94	0,92	0,86	0,98	0,83
0,77	0,92	0,91	0,87	0,71	0,70	0,99	0,95	0,96
0,76	0,95	0,80	0,84	0,71	0,59	0,85	0,89	0,95
0,91	0,93	0,95	0,91	0,84	0,96	0,88	0,98	0,86
0,93	0,95	0,83	0,99	0,89	0,90	0,79	0,95	0,84
0,73	0,88	0,90	0,95	0,63	0,64	0,98	0,94	0,95
0,84	0,97	0,90	0,82	0,82	0,94	0,87	0,86	0,73
